# Sex chromosomes drive gene expression and regulatory dimorphisms in mouse embryonic stem cells

**DOI:** 10.1186/s13293-017-0150-x

**Published:** 2017-08-17

**Authors:** Rachael J. Werner, Bryant M. Schultz, Jacklyn M. Huhn, Jaroslav Jelinek, Jozef Madzo, Nora Engel

**Affiliations:** Fels Institute for Cancer Research, Temple University School of Medicine, 3400 N. Broad St. PAHB Room 201, Philadelphia, PA 19140 USA

**Keywords:** Sex chromosomes, Transcriptome, Embryonic stem cells, Transcription factors, Epigenetic enzymes

## Abstract

**Background:**

Pre-implantation embryos exhibit sexual dimorphisms in both primates and rodents. To determine whether these differences reflected sex-biased expression patterns, we generated transcriptome profiles for six 40,XX, six 40,XY, and two 39,X mouse embryonic stem (ES) cells by RNA sequencing.

**Results:**

We found hundreds of coding and non-coding RNAs that were differentially expressed between male and female cells. Surprisingly, the majority of these were autosomal and included RNA encoding transcription and epigenetic and chromatin remodeling factors. We showed differential Prdm14-responsive enhancer activity in male and female cells, correlating with the sex-specific levels of *Prdm14* expression. This is the first time sex-specific enhancer activity in ES cells has been reported. Evaluation of X-linked gene expression patterns between our XX and XY lines revealed four distinct categories: (1) genes showing 2-fold greater expression in the female cells; (2) a set of genes with expression levels well above 2-fold in female cells; (3) genes with equivalent RNA levels in male and female cells; and strikingly, (4) a small number of genes with higher expression in the XY lines. Further evaluation of autosomal gene expression revealed differential expression of imprinted loci, despite appropriate parent-of-origin patterns. The 39,X lines aligned closely with the XY cells and provided insights into potential regulation of genes associated with Turner syndrome in humans. Moreover, inclusion of the 39,X lines permitted three-way comparisons, delineating X and Y chromosome-dependent patterns.

**Conclusions:**

Overall, our results support the role of the sex chromosomes in establishing sex-specific networks early in embryonic development and provide insights into effects of sex chromosome aneuploidies originating at those stages.

**Electronic supplementary material:**

The online version of this article (doi:10.1186/s13293-017-0150-x) contains supplementary material, which is available to authorized users.

## Background

The variation between males and females constitutes the largest phenotypic dimorphism in any given species [1]. In humans, this variation accounts for differences seen in the risk, incidence, and response to treatment for a plethora of diseases [2–4]. Examples include autoimmune diseases [5, 6], cancer susceptibility [7, 8], infectious disease incidence [9, 10], and cardiovascular disease presentation and risk [11, 12]. There is evidence showing transcriptional and epigenomic variability between men and women [13, 14]. The role of these differences in disease presentation and phenotype is unclear, but there is mounting evidence that gene regulation is linked to disease [15, 16], suggesting that the impact of sex on gene expression and epigenetic features is likely to be important [16, 17]. Sex hormones partially mediate sexual dimorphisms after gonadogenesis [18], but their action does not fully explain them [19].

The X and Y chromosomes encode multiple factors that have an important role in determining sexual biases in all tissues. This has been fully established in the four-core genotypes mouse model, in which the sex chromosome complement is uncoupled from gonadal sex [20, 21]. In this model, sexually dimorphic neural and behavioral traits were identified in adult mice and defects in neural tube closure at 10.5 days post coitum (dpc) were evident [21]. Differences in regulation of autosomal genes in male and female adult tissues were also shown in this system [22].

How soon do sexual dimorphisms appear in development? Differences between males and females start immediately after fertilization in both humans and mice. Male pre-implantation embryos develop more rapidly than female embryos, i.e., before gonadal sex differentiation, and, in mice, this difference persists at least up to day 9.5 of gestation [23]. Newborn males have a higher mortality rate than females [24]. Expression differences arising from the X and Y chromosomes directly or indirectly have been documented in mouse and humans [25–29]. The sex chromosome complement alone drives epigenome-wide differences in male and female murine embryonic stem (ES) cells [30–32]. These effects may have a lasting impact on developmental trajectories and disease risk [33, 34], even if transcriptional differences are not immediate.

We hypothesize that some sexual dimorphisms can be traced back to early embryogenesis because the presence of the sex chromosomes primes the genome differentially, leaving an epigenetic registry of sexual identity [35]. As a result, dosage differences, especially of transcription factors (TFs) and epigenetic complexes, can affect regulatory networks and determine differences in downstream developmental events. It has been shown that differential epigenetic marks can be latent, with effects on gene expression in later development, as tissue-specific signals become available.

It is widely accepted that various mouse strains are phenotypically different [36, 37]. Significant gene content variation exists between inbred mouse strains, including large deletions and amplifications [38], with the potential for biases in allelic gene expression [39]. To investigate the role of the sex chromosome complement on early transcriptional biases, we derived a panel of male and female hybrid mouse ES cell lines from reciprocal crosses between the C57BL/6 and CAST/EiJ mice and performed a genome-wide RNA expression comparison of 40,XX, 40,XY, and 39,X lines. This experimental design allowed us to gauge the impact of strain effects and focus on sex-specific expression. We found a substantial number of coding and non-coding RNAs that are differentially expressed between male and female ES cells, including RNAs encoding critical transcription factors and proteins involved in DNA methylation, histone modifications, and chromatin remodeling. Gene ontology analysis shows that male- and female-enriched RNAs code for proteins involved in distinct pathways, with male cells exhibiting a transcriptome more poised for differentiation than female cells. In addition, by comparing wild-type to 39,X cell lines, we identified genes dependent on either the X or the Y chromosome.

## Methods

The experimental strategy is outlined in Additional file 1: Figure S1.

### Embryonic stem cell derivation and culture

F1 hybrid blastocysts from natural matings were obtained at embryonic day 3.5 from reciprocal crosses of mouse substrains C57BL/6J and CAST/EiJ, denoted B and C hereafter; cell lines are designated as BC or CB, with the maternal line symbolized first. Blastocysts were cultured individually on mouse embryonic fibroblasts (MEFs) in gelatin-coated tissue culture plates and used to establish single, independent embryonic stem cell lines. Each line was maintained in ES cell culture medium (DMEM, 15% FCS, 1 mM sodium pyruvate, 2 mM L-glutamine, 1% nonessential amino acids, 0.1 mM 2-mercaptoethanol, and 1000 U/ml mouse leukemia inhibitory factor (LIF)) in 5% CO_2_ at 37 °C. Blastocysts hatched from the zona pellucida and adhered to the feeder layer after 2 days. After 4–6 days, the inner cell mass outgrowth was passaged using trypsin.

All mice were housed at the Fels Institute for Cancer Research and Molecular Biology animal facility. When required, euthanasia was performed by CO_2_ inhalation and animal sacrifice for blastocyst isolation was done by cervical dislocation. Animal studies for this project have been approved by the relevant institutional review board (protocol number 4472).

### Sex determination

ES cell lines were sexed by the presence or absence of *Sry* and a restriction digest of *Xist* using *Afl*III which selectively digests the C57BL/6 allele at rs225909365. After polyacrylamide gel electrophoresis (PAGE), resulting bands were quantified by densitometry analysis (GelAnalyzer2010 software) to confirm the presence of the maternal and paternal X chromosomes in female cells.

### Karyotyping

Sequenced lines were karyotyped at or near the passage from which RNA was obtained for library preparation to ensure chromosomal stability. Metaphase spreads were prepared according to previously published protocols [40]. A 100× oil immersion scope was used to obtained images of the spreads. Counting of > 100 cells was performed by separate individuals and compared for accuracy. The XY and XX cell lines possessed the diploid complement of 40 chromosomes in 85–90% of spreads. The two XO lines showed 39 chromosomes in 87–90% of spreads.

### RNA sequencing and Transcriptome analysis

Male, female, and aneuploid ES cells are denoted XY, XX, and XO hereafter for brevity. Fourteen (six XY, six XX, and two XO) early passage independent cell lines were used for RNA sequencing. Cell morphology and growth rates were consistent across lines at the time of collection. RNA isolation was performed using Roche’s High Pure RNA Isolation kit for the undifferentiated lines following two consecutive 60-min MEF-depletion steps. Isolated RNA was treated with TURBO™ DNase following the kit instructions from Ambion. Bioanalyzer electrophoresis as well a negative reverse-transcriptase and PCR (RT-PCR) were performed to ensure integrity and purity of the isolated RNA and absence of DNA contamination. One microgram of RNA from each line was used to generate a library according to Illumina TruSeq® Stranded Total RNA sample preparation guide. Lines were prepared in batches of six and then pooled and submitted for sequencing through a single flow cell at Fox Chase Cancer Center Sequencing Core using HiSeq 2500 single end reads of 50 bp. One to two percent PhiX was spiked in at the time of sequencing as an additional quality check. To identify differences in gene expression, sequenced reads were aligned to mm9 genome assembly using TopHat 2 software suite [41]. To determine differential gene expression, we used the Cufflinks software suite [42]. We used change *p* < 0.01 after correction for multiple testing (false discovery rate, FDR) for significantly differentially expressed genes. We used R software with Cummerbund packages for downstream analysis and visualization of the RNA-seq output including but not limited to hierarchical clustering and principal component analysis. For the heat maps, we isolated the most stably expressed genes by selecting the genes for which expression levels fell within the first quartile of mean normalized standard deviation between biological replicates. Assessment of pluripotency was performed using genes curated from published interactions, namely PluriNetwork [43] and PluriNet82 [44] with the addition of dynamically expressed genes. Cytoscape [45] was used for network visualization. Functional annotation was performed using DAVID (https://david.ncifcrf.gov/home.jsp) with a gene list cutoff of FDR < 0.05 [46, 47]. Visualization of functional output and gene ontology terms was generated through GO-plot using modified Fisher exact *p* value cutoff of < 0.1 [48].

### Quantitative PCR (qPCR) validation

Genes of interest showing differential expression were verified. The analysis was performed on cDNA generated using SuperScript™ II from RNA from multiple lines, including but not limited to the ones involved in the initial sequencing set. Relative gene expression was assessed using PowerUp SYBR Green Master Mix from Thermo Fisher and normalized to β-actin on Applied Biosystems StepOnePlus Real-Time PCR System. Some genes that showed no statistically significant difference in the training set were also tested to further confirm the validity of the RNA sequencing results (Additional file 2: Table S1).

### Luciferase assays

The reporter plasmids with enhancers responsive to Prdm14 and Trim24 cloned into a pGL3-*Oct4* promoter vector (Promega) were generously provided by Richard A. Young [49]. Transfections were performed using Lipofectamine 2000 (Invitrogen) according to the manufacturer’s recommendations. Testing was performed using three biological replicates from each cell line (XX, XY, and XO). ES cells were seeded onto a 12-well plate and then transfected with 800 ng of the reporter plasmid with or without the enhancer and 16 ng of the Renilla luciferase reporter (Promega) for 24 h at 37 °C. Firefly and Renilla activity were measured according to the instructions for Dual-Luciferase Reporter Assay System using a Glomax® Multi-Detection System (Promega). The relative luciferase activity was calculated by dividing Firefly luciferase by Renilla luciferase activity. Sequences were modified using primers designed on NEBaseChanger v1.2.5, and a protocol for mutagenesis using Q5® Hot Start High-Fidelity reaction components followed by digested with *Dpn*I and subsequent ligation and transformation into competent bacteria. Successful deletion or scrambling of the Prdm14-responsive motifs was verified by sequencing (Eurofins Genomics) and alignment to plasmids with Geneious version 6 (http://www.geneious.com) [50].

### Long non-coding (lnc)RNA interrogation

To evaluate differential long non-coding RNA expression between the male and female ES cells, sequences were aligned to mm10 and transcripts were identified with the NONCODE database (https://www.ncbi.nlm.nih.gov/pubmed/26586799). The lncRNA genes that overlapped with coding genes and were expressed from same DNA strand were filtered out from downstream analysis.

### Transcription factor motif analysis

The differentially expressed genes between male and female cell lines (FDR ≤ 0.05) were used for de novo motif discovery with parameters set for 5000–1000 bp upstream of the transcriptional start site in HOMER (http://homer.ucsd.edu/homer/) (Additional file 3: Table S2).

### Allele-specific RT-PCR

After reverse transcription, the *Cdkn1c* transcript was amplified using Ruby Taq Master Mix (Affymetrix - #71191). Following PCR, restriction digest was performed with a *Taq* *I* (New England Biolabs) and products were run on 7% PAGE [51]. The primers for *Cdkn1c* were 5′-CGGACGATGGAAGAACTCTGG-3′ and 5′-TATACCTTGGGACCAGCGTACTCC-3′.

## Results

### Sex-specific expression in ES cells

To determine whether differential expression occurs during early development, we used mouse ES cells cultured in serum and LIF, denoted as serum/LIF, as a model for peri-implantation embryos. This medium was preferred because culture of ES cells with inhibitors of Mek1/2 and Gsk3β in conjunction with LIF (2i/LIF) leads to widespread reduction in DNA methylation, including erosion of methylation of imprinting control regions (ICRs), compromising the ability of the cells to contribute to development [30, 52–55]. We derived stable male (XY), female (XX), and 39,X (XO) ES cell lines from blastocysts originated by reciprocal crosses between C57BL/6 (B) and CAST/EiJ (C) mice (designated as BC and CB, with maternal strain indicated first; male cells, BCM or CBM; and female cells, BCF or CBF). We analyzed the transcriptomes by RNA sequencing (RNA-seq) from three early passage cell lines of each cross and sex and two early passage XO cell lines derived from a BC cross (denoted BCO). We detected 14,766 transcripts and compared the data to quantitatively assess the differentially expressed genes. Alignment and transcript coverage was similar between all samples assayed.

**Fig. 1:**
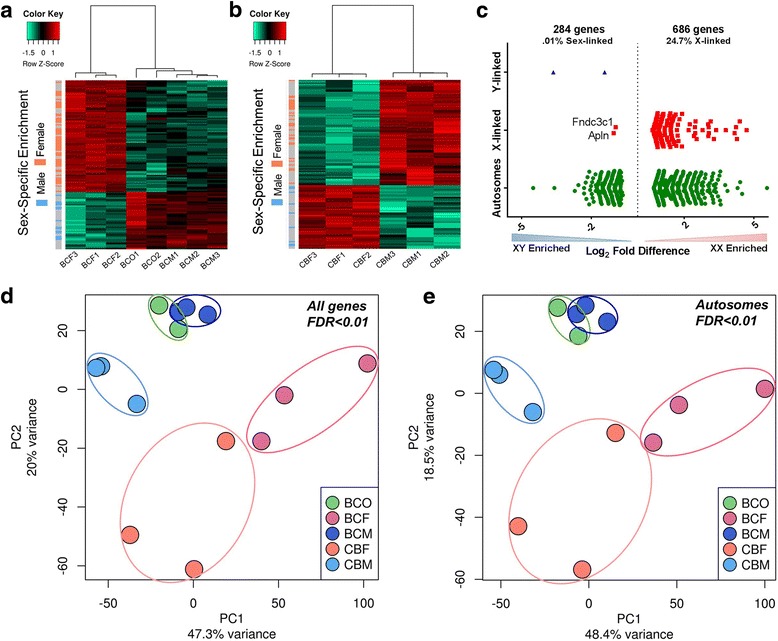
Identification and biological replication of sexually dimorphic genes in mouse ES cells. **a**, **b** Transcripts were defined as dimorphic from RNA-seq data using a genome-wide corrected FDR < 0.01 and standard deviation between biological replicates of less than 10%. Genes with sex-specific biases independent of strain are indicated on the *y*-axis. Sexually dimorphic expression in BC ES cells (**a**) showed clustering of the two XO lines with the three XY lines, separately and distinctly from the three XX lines. CB ES cells (**b**) showed clustering by sex chromosome complement with the three XY grouped together separately from the three XX lines. **c** Chromosomal distribution of sexually dimorphic genes when comparing all six XX and six XY ES cells at FDR < 0.01. **d** Principal component analysis (PCA) of differentially expressed genes at FDR < 0.01 from RNA -seq of ES cells shows distinct clustering of each cell line with close association of lines of the same strain and sex, with the exception of the two XO lines which highly overlap with the three XY lines of the same cross (BC). **e** Exclusion of sex chromosome-linked expression in the PCA at FDR < 0.01 shows maintenance of segregation of the ES lines based on strain and sex affirming that autosomal expression alone accounts for these biases

We first compared transcriptomes between sexes within each cross to determine sex-specific differentially expressed genes. The ES cell lines showed hierarchical clustering by sex chromosome complement (Fig. 1a, b). Evaluation of the chromosomal distribution of the differentially expressed genes shows that the differences between XY and XX lines are primarily derived from expression originating from autosomal regions (Fig. 1c). The XO lines (BCO) more closely resembled the XY lines (BCM) than the XX lines (BCF) of the same cross, which is illustrated in the principal component analysis generated using differentially expressed genes at a FDR < 0.01 (Fig. 1d).

Because close to 25% of the differential gene expression originated from the sex chromosomes, we performed a principal component analysis excluding sex-linked genes at a FDR < 0.01 to evaluate if the expression levels from the sex chromosomes alone were driving the difference seen in the hierarchical clustering. Interestingly, autosomal gene expression differences alone are enough to segregate the ES cells by sex, with Fig. 1e showing segregation by cross and then by sex chromosome composition with the CBM and BCF lines showing the largest degree of separation. This result illustrates that the sex chromosome composition influences autosomal gene regulation and expression in a differential and detectable manner.

**Fig. 2:**
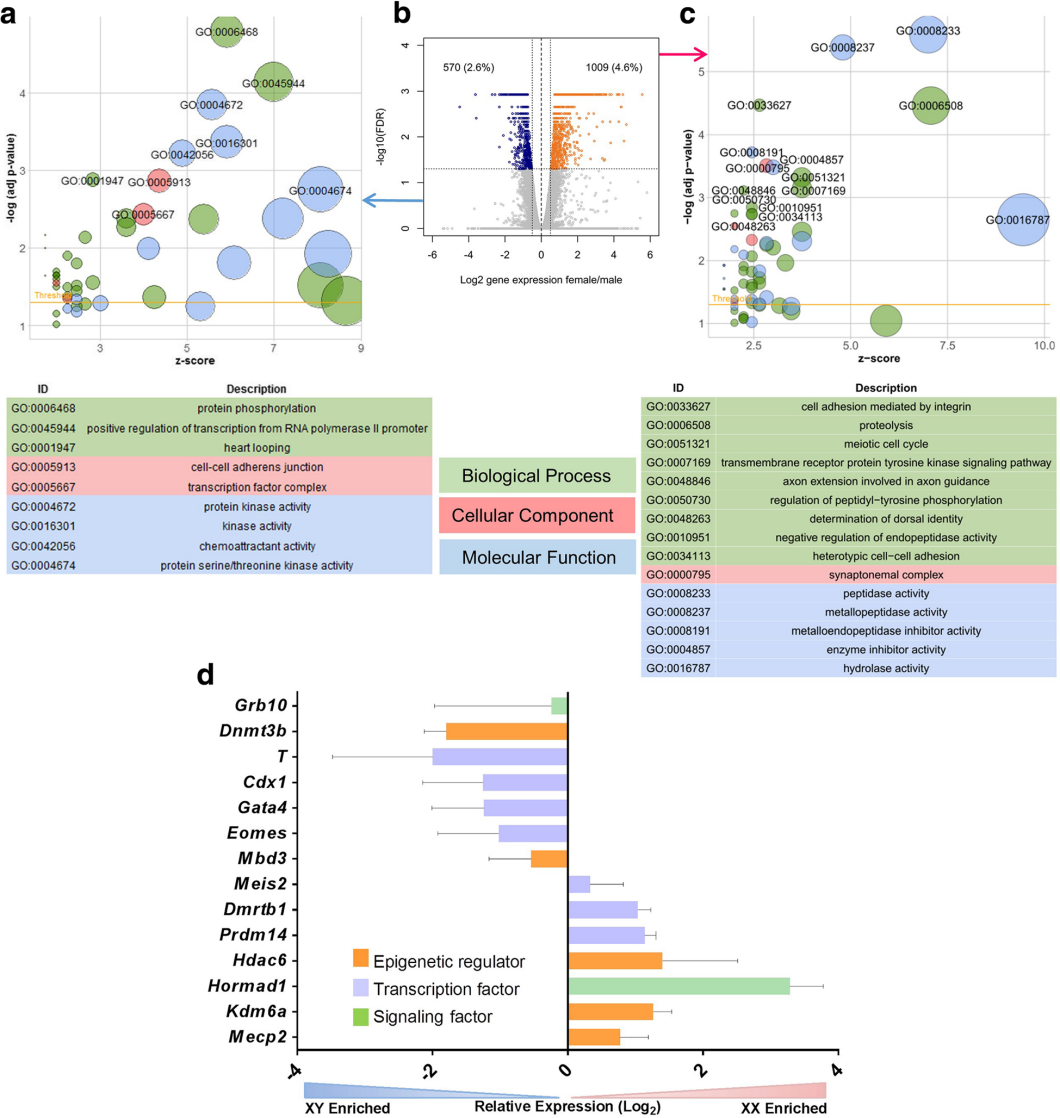
Gene ontology of differentially expressed genes between XY and XX ES cells. **a**, **c** Upregulated biological process, molecular function and cellular components between XY (**a**) and XX (**c**) ES cells. The XY lines had increased expression of genes related to early development (heart looping) and kinase activity. The XX lines showed increased expression of meiotic cell cycle-associated genes and peptidase activity. x-axis: *z*-score of associated terms, which indicates degree of upregulation; *y*-axis: significance of term. **b** Volcano plot showing selection of the differentially expressed genes between XY and XX lines at FDR < 0.05 (blue dots, higher in males; orange dots, higher in females). **d** qPCR validation of genes detected in the RNA sequencing dataset illustrating a sex-biased expression pattern of transcription factors, epigenetic enzymes and key signaling factors

A limitation of some mouse studies is the use of a single inbred mouse line to identify sex-specific effects. Polymorphisms are capable of skewing gene expression and as there is mounting consensus for the use of multiparent strains to more closely approximate human diseases [56], we compared all XY to all XX ES lines. At FDR < 0.05, we detected over 1500 genes differentially expressed between XY and XX lines (Fig. 2b). Over a thousand genes were more highly expressed in XX cell lines. XY lines were enriched for epithelial development and differentiation as well as cell-cell junctions (Fig. 2a). Interestingly, genes with higher expression in female ES cells were enriched for terms associated with cell cycle, meiosis, nuclear chromosome, and microtubule organization (Fig. 2c). These results suggest that male cells are more poised for lineage determination even before differentiation induction. It also supports the hypothesis put forth previously that the double X chromosome dosage stabilizes pluripotency [27].

To trace regulatory differences in XX and XY cells, we focused on transcription factors and epigenetic and chromatin remodeling factors (TFs and ERFs, respectively). At FDR < 0.01, 49 TFs were enriched in XX cells, including 13 X-linked factors: *Klf8*, *Hmgb3*, *Rhox1*, *Rhox9*, *Elf4*, *Zfp182*, *Zfp449*, and *Aff2*, among others. Autosomal TFs enriched in XX cells included *Dmrtb1*, *Meis2, Prdm14*, and interestingly, 11 genes of the *Hox* (homeobox) family. ERFs more highly expressed in XX cells (22) included *Mecp2*, *Apobec2*, *Parp3*, and *Kdm6a* (previously reported [26]) (Additional file 4: Table S3).

In XY cells, 59 TFs had significantly higher levels compared to XX cells. These included *Eomes, Prdm6*, *Gata4, Mef2b*, and *Ybx*2. XY cells had higher RNA levels of 20 ERFs, including *Dnmt3a, Dnmt3b*, *Hdac5*, *Mbd3*, *Cxxc1*, and *Uty (Kdm6c)* (Additional file 4: Table S3). We evaluated expression of a subset of genes of interest including TFs, ERFs, and signaling molecules with qPCR assays, to confirm the RNA sequencing data (Fig. 2d). These genes were selected based on their associations with key cellular pathways and processes.

### Regulatory network for pluripotency in XX and XY ES cell lines

**Fig. 3:**
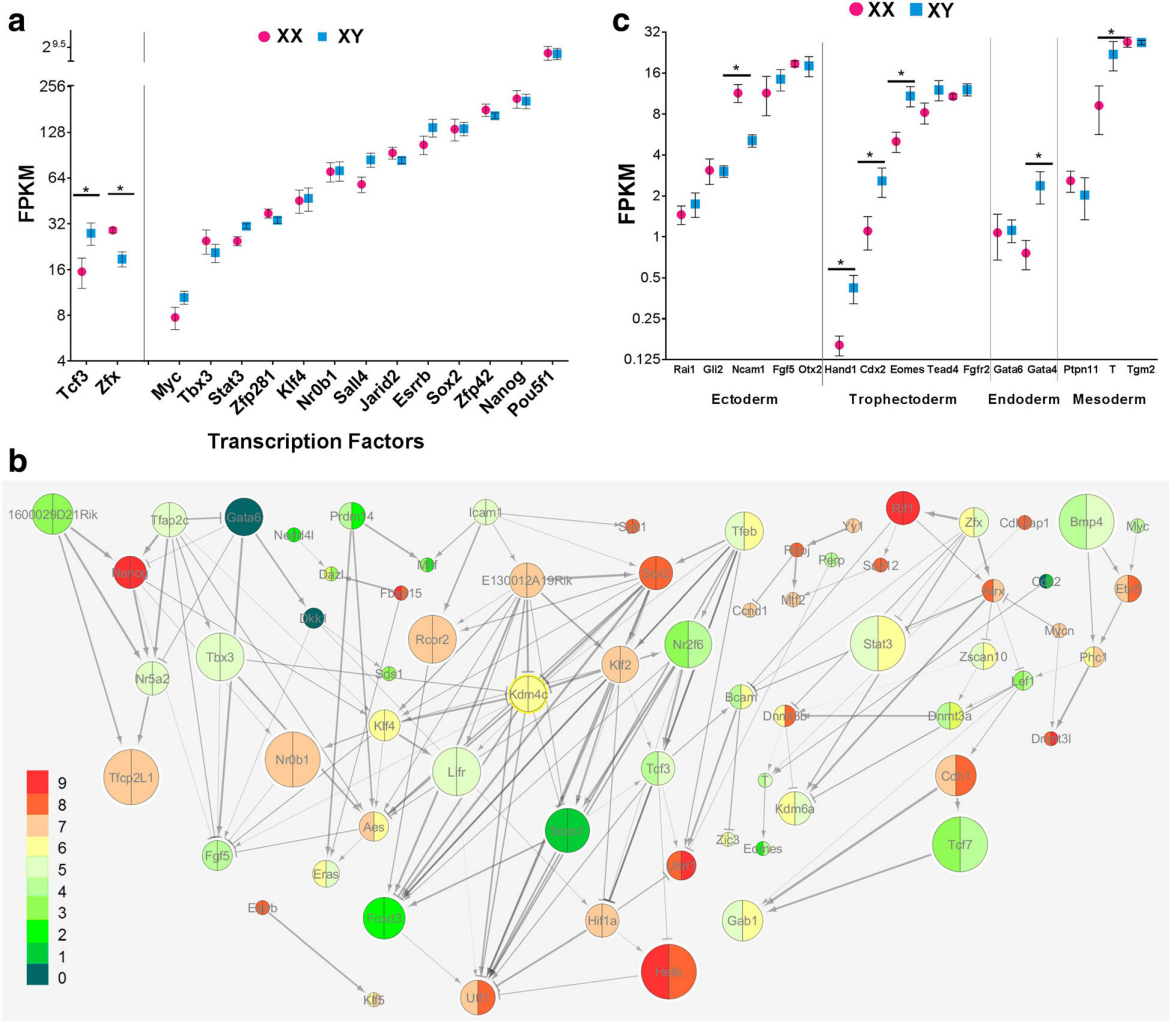
Pluripotency and lineage-specific factors in XX and XY ES cells. **a** Fragments per kilobase per million (FPKM) values from RNA sequencing of six XX and six XY ES cells showing expression of 15 of the minimal network of pluripotency transcription factors. Two transcription factors were differentially expressed between the XX and XY cell lines: *Tcf3* was 1.8-fold greater in XY, and *Zfx* was 1.5-fold higher in XX. *FDR < 0.05*.* **b** In the expanded pluripotency network, there are detectable and distinct differences in expression of key genes between XX and XY cells. Node size reflects the clustering coefficient and line thickness denotes the statistical significance of the interaction with arrows indicating a positive interaction and T bars indicating inhibition. Colors represent relative expression levels in RPKM for XX (left) and XY ES cells (right). **c** Differences in expression of lineage-specific markers. Note that expression levels were lower compared to levels of pluripotency-associated genes. The ectodermal lineage-specific marker *Ncam1* was 2-fold higher in XX lines. The XY cells were enriched for *Hand1*, *Cdx2*, *Eomes*, *Gata4*, and *T* when compared to the XX lines with fold change of 2.6, 2.5, 2.4, 3.7, and 2.5, respectively. *FDR < 0.03

The XY ES cell lines exhibited enrichment for pathways associated with epithelial development and morphogenesis and seemed more poised for differentiation. We assessed the transcriptomes of all XX and XY ES cell lines to confirm that they maintained expression of the genes commonly associated with the minimal pluripotency network [57, 58]. All of the pluripotency-associated transcription factors (TFs) were expressed. However, two of them were differentially expressed between male and female cells, with *Tcf3* expressed at higher levels in the males and *Zfx* higher in the females (Fig. 3a). As an X-linked gene, *Zfx* was expected to be 2-fold higher in female cells. Although the Y chromosome homolog, *Zfy*, could compensate this imbalance, it was not expressed in male cells. We investigated the regions upstream of *Zfx* and *Tcf3* to see if they are regulated by TFs that are also differentially expressed by combining the histone modification profiles typical of enhancers from ENCODE data at the USCS genome browser (http://genome.ucsc.edu/) [59–63] and the conservation by comparative genomics from the dCODE website (http://www.dcode.org/) [64, 65]. The most likely enhancer for *Zfx* has binding sites for Mef2 TFs, one of which (*Mef2b*) was expressed at higher levels in female cell lines (Additional file 5: Figure S2A). A candidate enhancer for *Tcf3* has binding sites for Gata4, present at higher levels in the male cells (Additional file 5: Figure S2B).

Because we saw differences in the minimal network, we decided to assess additional genes curated from published interactions, namely PluriNetwork [43] and PluriNet82 [44]. Focusing on dynamically expressed genes and the core regulators of *Nanog* and *Sox2*, we generated a network of 90 genes. From this set of genes, we performed pathway analysis using the RNA-seq data generated from all of the XX and XY ES cell lines and found statistically significant interactions involving 69 of the genes, FDR *<* 0.05 (Fig. 3b).

### Differential expression of *Prdm14* in ES cell lines leads to differential enhancer activity

Prdm14 plays a key role in pluripotency [66, 67], as well as in X chromosome reactivation in reprogramming [68]. We observed that *Prdm14* had significantly higher expression levels in XX ES cells (Fig. 2d). Prdm14 generally recognizes DNA motifs distant from transcriptional start sites, overlapping with candidate regulatory sequences [69], including enhancer clusters that drive high-level expression of genes that are key regulators of cell identity [49, 70]. To see if higher *Prdm14* levels correlated with expression of its targets, we checked the overlap with pre-existing data on Prdm14-regulated genes [67, 71]. Indeed, genes repressed by Prdm14 such as *Dnmt3b, Cdkn1c*, *FoxA2*, and *Gata4* exhibited lower expression in XX cells, whereas activated genes, such as *Prdm14* itself, *Meis2*, *Dazl*, *Mitf*, and *Zeb1* were more highly expressed. A substantial number of genes from the knockdown experiments, however, did not correlate with the higher *Prdm14* levels, possibly because of the differences in culture media. Binding data from tagged *Prdm14* ChIP-seq experiments, however, confirmed that Prdm14 is present in the vicinity of *Dnmt3a, Dnmt3b, Gata4*, and *Meis2* [67].

**Fig. 4:**
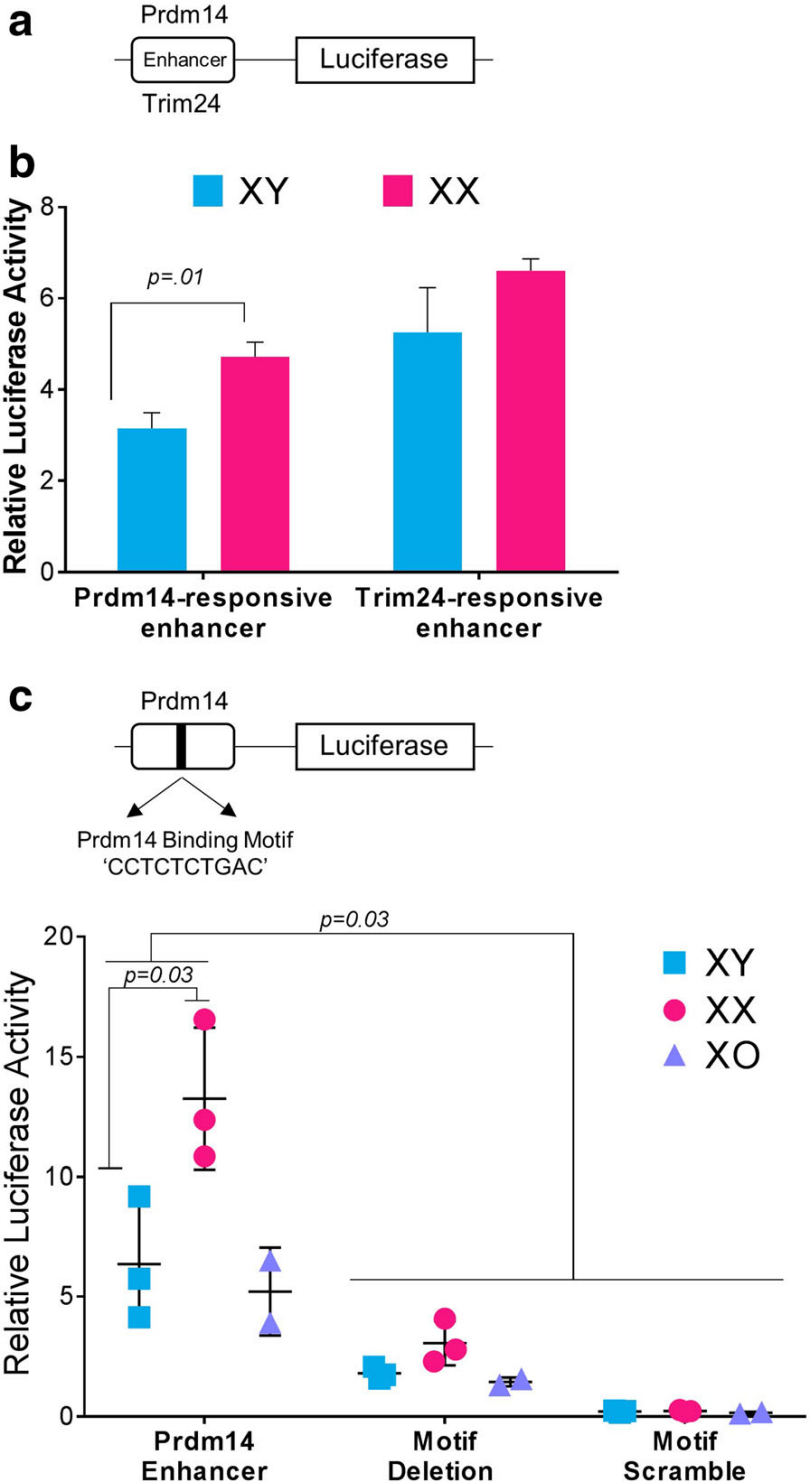
Prdm14-associated enhancer response correlates with *Prdm14* dosage. **a** Schematic of vectors used in luciferase assays. **b** XX and XY ES cells were each transfected with vectors containing either a Prdm14- or Trim24-responsive enhancer. Luciferase activity was higher in XX ES cells for the Prdm14-responsive enhancer. *Trim-24* mRNA levels were equal in XX and XY ES cells and the Trim24-responsive enhancer showed no difference in enhancer activity. Error bars represent SEM. Samples were normalized to an empty vector control. **c** Top, schematic of the luciferase plasmid and the binding motif for Prdm14. Both the deletion and scrambling of the motif reduced the sex-specific differences and decreased overall luciferase activity in all biological replicates

To assess if *Prdm14* dosage differences in XX and XY ES cells correlated with differential enhancer activity, we performed luciferase assays in three biological replicates of XX and XY ES cells with vectors containing known enhancers responsive to Prdm14 and Trim24 [49] (Fig. 4a). Luciferase activity of the plasmid containing the Prdm14-responsive enhancer was higher in the XX ES cells (Fig. 4b), highlighting that there are sex-specific differences in autosomal gene regulation very early in development. This is the first report of such biased enhancer response. Using the consensus binding motif derived from ChIP studies [67] and an algorithm for binding potential (Geneious version 10.0.2, http://www.geneious.com) [50], we identified and tested three potential Prdm14 motifs in the enhancer. Deletion and scrambling of one of these motifs (Fig. 4c) greatly reduced luciferase activity and abolished the sex-specific differences. *Trim24* was expressed similarly between the XX and XY ES cell lines and served as a negative control. Enhancer activity in XO ES cells mimicked the XY cells. These results suggest that, in addition to chromosomal environment, TF dosage contributes to the expression outcome.

### Differentiation markers in XX and XY cells

The variation in expression of pluripotency-associated genes prompted us to look at markers of differentiation. We evaluated the data using the 15 lineage-associated markers derived from the ESCAPE database [72] and found that XX lines were enriched for the ectodermal lineage marker *Ncam1* (2-fold), while the XY and XO lines were enriched for trophectoderm markers *Hand1*, *Eomes* and *Cdx2*, endodermal marker *Gata4*, and the mesodermal marker *T* (*Brachyury*)(FDR *<* 0.04) (Fig. 3c).

We considered whether genes were differentially expressed in male and female cells because the XX cells are slightly delayed, as reported in a previous study [27]. If the observed differences were due to an offset in developmental stage, we would expect genes more highly expressed in male cells to be more typical of differentiated ES cells*.* Inspection of published data on ES cell differentiation into cardiomyocytes [73] showed that this was not the case. For example, *Pou3f1*, *Cdx1*, *Mycn, Mef2b*, and *Dnmt3b,* among others, are higher in male cells, but downregulated upon differentiation. Thus, higher expression levels of these genes are specific to undifferentiated XY ES cells. If the expression pattern in female cells is due to developmental delay, genes expressed more highly relative to XY cells would be downregulated upon differentiation. On the contrary, genes expressed more highly in females, such as *Meis2*, *Zeb1*, *Apobec2*, and *Mecp2*, were upregulated during differentiation.

Furthermore, we compared our data to the list of genes with delayed XX expression obtained by microarray analysis in one male and one female ES cell line upon differentiation into embryoid bodies [27]. Only 104 (8%) out of 1284 genes from that list overlapped with our list of RNAs enriched in XX cells. These data confirm that the vast majority of expression biases we observed are not due to stage-specific patterns but are *bona fide* sexual dimorphisms. Our results also suggest that analysis of six cell lines for each sex by RNA sequencing is more powerful and sensitive, allowing identification of a greater number of differentially expressed genes.

### X chromosome-linked expression

X chromosome inactivation (XCI) in females is a crucial event during mammalian development [58, 74]. In ES cell lines maintained in serum/LIF conditions, a minimum of 94% of the cells consistently show two active X chromosomes [27, 75]. Thus, many X-linked genes are expected to be expressed 2-fold higher in female than in male cells. Not surprisingly, 25% of female-enriched genes were on the X chromosome. Unexpectedly, however, close to 35% of all the X-linked genes with higher expression in XX cells showed more than a 2-fold difference. One of these, *Srpx,* which was 5.6-fold higher in the XX cells, is a tumor suppressor gene that is downregulated in a number of human malignancies, including prostate, colorectal, and neuroendocrine cancers [76, 77]. Other X-linked genes with RNA levels more than 2-fold higher in female cells were *Rhox1*, *Gm9*, and a large cohort of genes from the *Xlr* family associated with immune-related processes. These findings suggest that there is an additional layer of regulation directing the expression of these genes. Interestingly, *Xlr3c* and *Xlr3a* are clustered, as are *Rhox1* and *Gm9*, which may indicate common regulatory mechanisms (Additional file 6: Table S4).

Also surprising was the finding that close to 50% of all X-linked genes expressed in ES cells had equivalent levels in both male and female cell lines, before X chromosome inactivation had occurred.

Two X-linked genes were expressed at higher levels in the male cells, with FDR < 0.01 (Fig. 1c). One of these, *Apln*, has highly conserved regions upstream of the promoter with chromatin features consistent with enhancer activity. Analysis of these sequences yielded motifs for TFs Hand1 and Lef1, both of which were expressed more highly in male ES cells (Additional file 5: Figure S2B).

**Fig. 5:**
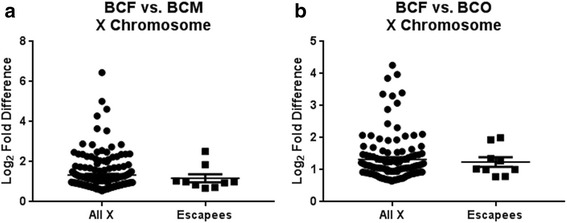
Expression of known X chromosome escapees compared to the expression of other X-linked genes that showed enrichment in XX ES lines using a Mann-Whitney test. **a** Comparison of expression levels of genes escaping XCI between BCF and BCM lines shows no statistical difference (*p* = 0.6420). **b** Expression levels of X-linked genes was compared between BCF and BCO, showing no statistical difference (*p* = 0.9747)

In mice, 3% of genes escape X chromosome inactivation (XCI) and remain active, depending on the tissue [78, 79]. We asked if those genes are more highly expressed than other X-linked genes in XX lines than in XY lines before ES cell differentiation, perhaps counteracting the chromosome-wide silencing mechanism. We did not find that to be the case, suggesting that, if those genes are pre-marked for later escape, other mechanisms are involved (Fig. 5a; Additional file 7: Table S5). Whether these genes maintain comparable levels to their XY counterparts after XCI is still under debate [80–82]. Escapees are also not overexpressed in XX versus XO ES cells (Fig. 5b).

### Identification of promoter signatures for differentially expressed genes

The promoter architecture of XX and XY differentially expressed genes were analyzed for enrichment of TF motifs from the transcriptional start site (TSS) − 1500 to + 500 and − 5000 to + 500 bp (Additional file 3: Table S2). For genes exhibiting higher expression in XX ES cells, motifs for Arid5a, Nkx2–5, HoxA2, Smad3, Tead2, HoxA5, Mecom, and Zfx, among others, were enriched (*p* ≤ 10^− 8^). In fact, RNA levels for *HoxA2, HoxA5, Mecom*, and *Zfx* were higher in XX cells. For genes with higher expression in male cells, motifs for Runx1, Foxq1, Hic1, Prrx2, and Tcf3, among others, were more prominent (*p* ≤ 10^− 8^). However, only *Tcf3* was higher in XY cells. Many TFs have overlapping and redundant binding potential, so combinatorial effects as well as accessibility could be contributing to the sex-biased expression patterns.

### Detection and differential expression of long non-coding RNAs

**Fig. 6:**
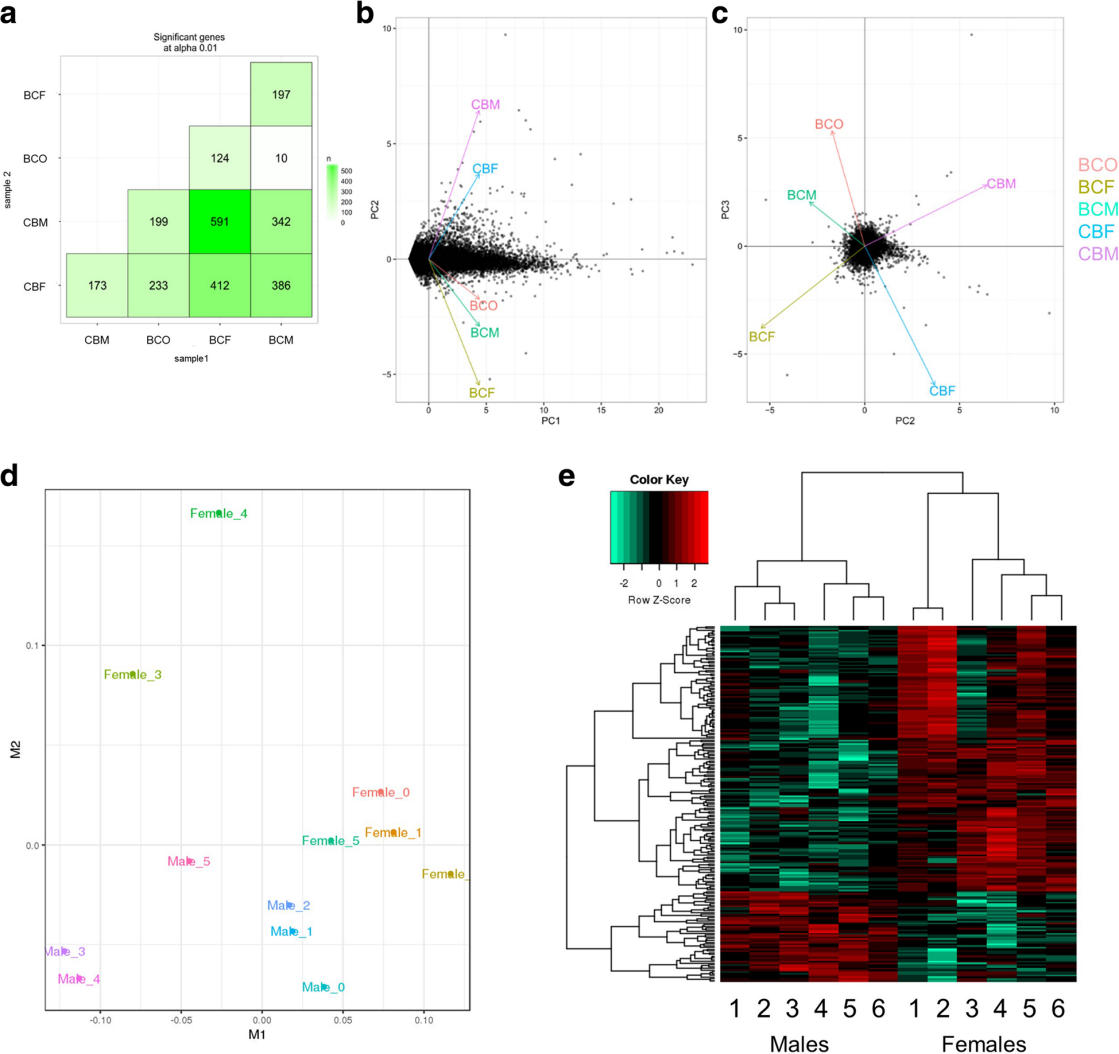
Identification and biological replication of sexually dimorphic lncRNAs identified in ES cells. Transcripts were defined as dimorphic from RNA-seq data using a genome-wide FDR (alpha) < 0.01. **a** Sexually dimorphic expression between all conditions tested. The highest degree of variation was seen between BCF and CBM which 591 differentially expressed lncRNAs. **b** Principal component analysis of lncRNAs shows segregation of cells based on strain of origin. **c** Plotting principal component 2 against component 3 shows segregation based on sex chromosome complement with BCO aligning more closely to BCM than to BCF, corresponding to the analysis performed using coding genes. **d** Multidimensional scaling plot showing clustering of the XY and XX lines. M1 corresponds to strain of origin, with M2 showing separation based on sex chromosome composition. **e** Heatmap showing dimorphic lncRNA expression and clustering of samples based on sex chromosome composition (FDR < 0.01)

The RNA-seq data was assessed for differences in expression of lncRNAs. Comparison between all samples assayed showed the highest degree of variation between BCF and CBM, which coincides with the variation seen in coding genes (Fig. 6a). Comparison of lncRNA expression showed segregation first by direction of cross followed closely by sex chromosome complement (FDR < 0.01) (Fig. 6b, c). As with coding genes, the XO cell lines (BCO) were more similar to XY lines (BCM) of the same cross type, with only 10 lncRNAs showing differential expression (Fig. 6d). Of note, we detected more differences between BCM and BCF (412 lncRNAs) than between CBM and CBF (173 lncRNAs) (Fig. 5a).

To assess sex-specific expression of lncRNAs, we compared all six XY to all six XX ES cell lines. Over 200 lncRNAs were differentially expressed (FDR < 0.05). As with the coding genes, we saw separation based on sex chromosome composition (Fig. 6e).

### Imprinted loci: expression and regulation

**Fig. 7:**
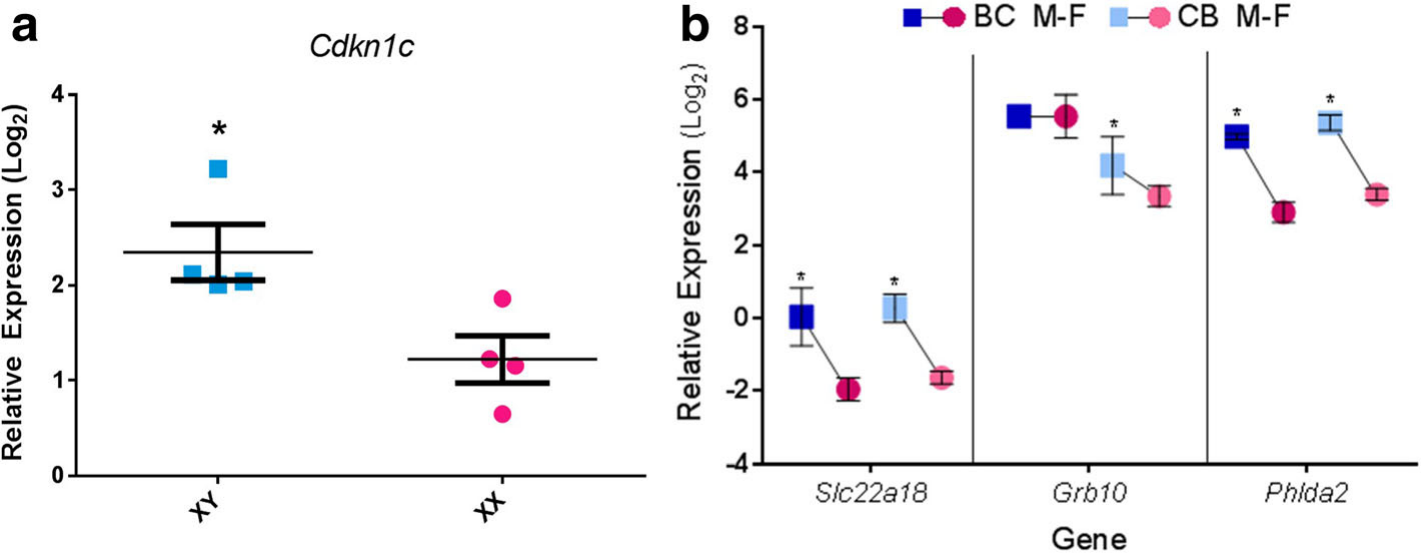
Imprinted gene expression. **a** Differential expression of imprinted gene *Cdkn1c* validated in four XX and XY lines. Error bars represent SEM. **b** Relative expression levels three additional imprinted genes. Comparisons are within each cross. Expression levels of these imprinted loci were generally greater in males than in females. Asterisk denotes significance (FDR < 0.01)

Unexpectedly, some imprinted genes exhibited higher expression in the male cell lines (Fig. 7) including the maternally expressed *Cdkn1c*, *Phlda2*, and *Slc22a18*. To see whether this was due to loss of imprinting, we performed allelic expression assays and observed monoallelic expression from the appropriate parent of origin allele for the BC cells, i.e., C57BL/6 allele, and maternally biased expression for the CB cells (Additional file 8: Figure S3). Loss of methylation is unlikely to occur in male cells, since they are more highly methylated than XX ES cells [83]. In the case of *Cdkn1c* and *Phlda2*, the higher expression could be due to the male cells being slightly ahead of the female cells in the lineage determination path, since both of these genes are greatly induced upon differentiation, but this is not the case for *Slc22a18*. Because these genes are clustered, a more likely explanation is that they are subject to an additional regulatory mechanism active on the maternal allele, directly or indirectly dependent on sex chromosome composition. *Grb10* RNA levels, on the other hand, were only higher in the male cells of the CB cross. Strain differences present an opportunity to discover the as yet unknown enhancers directing expression of these genes during development.

### Comparisons with currently published datasets

We compared our results with a recent publication that analyzed expression differences between male and female ES cells across different developmental stages using single-cell RNA sequencing (scRNA-seq) [75]. We used homologous datasets, i.e., cells cultured in serum/LIF and the comparable cross, i.e., C57BL/6 × CAST/EiJ, because only this unidirectional cross was analyzed in that study. Due to the inherent noise and abundance of zero entries within the data matrix, a common occurrence within scRNA-seq analyses [84, 85], we compared the concordance of direction of gene expression between the two sexes in both datasets with a sign test. Comparison of the differentially expressed genes within our dataset at FDR < 0.05 using an exact binomial test showed a high degree of correlation at a *p* value of 2.2e^− 16^ and a 95% confidence interval (CI) of 0.58 to 0.60. To more closely examine the correlation, we compared expression of the 14 genes assessed by qPCR (Fig. 2d) with the single-cell data (Additional file 9: Figure S4) and found a high degree of correlation *r* = 0.78 (*p* = 0.001). We also performed a sign test on this subset of genes revealing a strong concordance of expression with a *p* value of 0.002 and 95% CI of 0.66 to 0.99. Of note, the sign test for *Prdm14* agreed with our data, confirming that *Prdm14* expression shows a sex-specific bias.

Single-cell RNA-seq [75] was also performed on sexed post-implantation embryos (5.5 dpc). We analyzed the published dataset to determine which genes had a sex-dependent bias (*p* < 0.01). We then compared with our ES cell data from the comparable cross to see if there were genes that maintained their sex biases. The overlap in female-biased genes between the two datasets was significant by hypergeometric testing (73 genes, *p* = 0.007), but only 10 male-biased genes overlapped (Additional file 10: Table S6). Among them were the Y-linked genes *Ddx3y* and *Uty* and interestingly, *Dnmt3b.* This shows that female ES cells and post-implantation embryos are more similar than the male counterparts, suggesting that the female 5.5 dpc embryos are still delayed at that time point.

### Sex chromosome-dependent expression

We analyzed two XO cell lines that arose spontaneously during ES cell line establishment, both of which were derived from a BC cross (designated BCO)*.* Both aneuploid cell lines expressed all the genes associated with the minimal pluripotency network [57, 58]. BCO lines were most similar to BCM cell lines, with only 116 genes showing statistically significant differences in expression (FDR < 0.05). As was the case for XY cells, XO lines had higher expression of *Tcf3* than XX cells, but in addition, they had higher levels of *Sall4* and *Esrrb*.

To identify if similar regulatory mechanisms were contributing to the differential expression in BCM and BCO when compared to BCF, we plotted the chromosomal distribution of the shared and unique enriched sets of genes for the male and XO cell lines (Additional file 11: Figure S5). The average proportion of overlap of enriched genes per chromosome relative to the female cells was 48%, with several chromosomes showing greater than 63% overlap, notably chromosome 1, chromosome 14, and chromosome 16. We quantified the variation in expression based on whether the gene was uniquely enriched in BCO or shared with BCM relative to BCF. The genes common to both sets showed statistically higher enrichment than those that did not (*p* = 7.3e− 7), suggesting that the lack of a second X chromosome is the predominant driver of expression differences in this model.

**Fig. 8:**
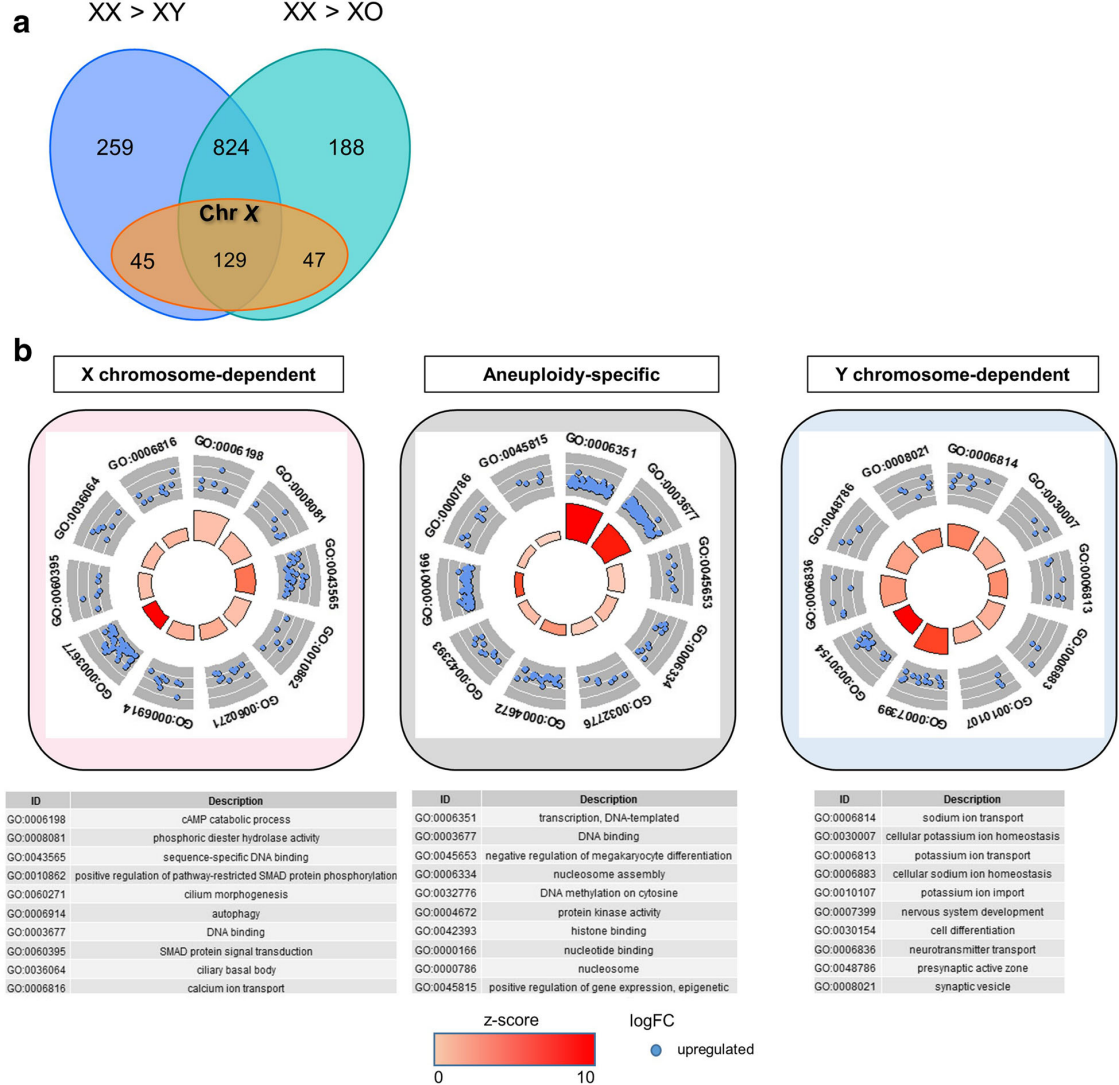
Differential gene expression analysis across karyotypes. **a** Expression comparisons between XX, XY, and XO ES cell lines, showing the extent of shared differences in pair-wise comparisons for X-linked and autosomal genes. **b** Gene ontology of genes showing X chromosome dependency, Y chromosome dependency, and aneuploidy-specific effects. Genes showing X chromosome dependency, aneuploidy-specific effects, or Y chromosome-dependency fall within unique ontology terms. Visualization performed using the GO-plot package in R. The outer circle shows a scatter plot of the log-fold change for the assigned genes within each distinct gene ontology term. Selection for enrichment resulted in blue dots only, which indicate upregulation. The inner circle is a bar plot with the height of the bar illustrating the *p* value of the term and the color representing the *z*-score, which evaluates the direction of the fold change of each of the genes within the assigned term and provides a relative scale for enrichment

To better understand the impact of the sex chromosome aneuploidy, we did two-way comparisons between XX, XY, and XO cell lines and identified differentially expressed genes that were exclusive to each pair (Fig. 8a). Eight hundred twenty-four genes were upregulated in XX relative to both XO and XY cells: 129 of these are X-linked. Two hundred thirty-five genes were more highly expressed exclusively in XX relative to XO cells, suggesting RNA levels directly or indirectly dependent on the presence of the two X chromosomes. We expected most of these to be X-linked, but only 45 (20%) are on the X chromosome, highlighting that the bulk of the transcriptional differences are downstream of the sex chromosomes and reflect their regulatory effect on the autosomes. Eight TFs and four ERFs are among the differentially expressed genes, as well as eight genes associated with Turner syndrome (*PROCR, NR3C1, INSR, APOB, BMP15, F8, IL6,* and *WAS*) [86–88] and two genes that are haploinsufficient in humans (*RAD50* and *SLC33A1*) [88]. Neither the Turner-associated nor the haploinsufficient genes are overexpressed in male versus female ES cells, indicating that they are specific for the aneuploidy. Additionally, two imprinted genes, the maternally expressed *Rian* and *Grb10* had higher levels in XX versus XO lines.

X chromosome dosage also exerts strong repressive effects: hundreds of genes were upregulated in XO cells relative to XX lines. Four hundred thirty-four genes were more highly expressed in XO uniquely versus XX cell lines. Fifty-five TFs and 25 ERFs were among them. These genes showed enrichment for 50 ontology terms at FDR < 0.05 which included regulation of biosynthetic processes as well as terms associated with epigenetic regulation involving nucleosomes, chromatin, and DNA binding (Fig. 8b). Included in this group are four genes commonly associated with Turner syndrome (*WNT4*, *MTHFR*, *FGFR3*, and *MEN1*) [88]. The Turner-associated genes are not higher in XY versus XX cells, indicating that X chromosome dosage alone does not explain their derepression.

XO cell lines had very similar expression profiles to XY cells. Consistent with this, only 15 genes were more highly expressed in the XY cells, one mapping to the Y chromosome (*Eif2s3y*). Based on our data, we infer that genes that were higher in XY than in both XX and XO cells (294 genes) are dependent on the presence of the Y chromosome.

## Discussion

Sex determination in mammals is classically defined as the developmental decision of whether the gonad becomes a testis or an ovary [89, 90]. There is long-standing evidence, however, that phenotypic differences between males and females precede the formation of the gonads [25, 35].

The findings presented in this study demonstrate that sexually biased genes expressed in embryonic stem cells integrate distinct regulatory networks very early in development. Transcription factors (TFs) and epigenetic and remodeling complexes (ERFs) are among the hundreds of differentially expressed genes, including TFs belonging to the pluripotency network. We found that an enhancer responsive to Prdm14 is more active in female ES cells, corresponding to the higher *Prdm14* levels. We also detected more than 300 differentially expressed lncRNAs. Only a small fraction of differences can be accounted for by a developmental delay imposed on female cells by the need to inactivate an X chromosome. We also analyzed genes that were differentially expressed between 40,XX and 39,X ES cells and found multiple differences, some of which may account for phenotypes observed in Turner syndrome. These findings advance on previous work [26–28] by defining a signature for mouse ES cells in conditions that mimic the peri-implantation stage, by analyzing six cell lines for each sex and by highlighting the differences with monosomic X cell lines. In addition, we detect genotype-sex interactions which will provide a rich resource for understanding the effects of genomic variation and the need for canalizing the developmental processes essential for both sexes [91].

### Sex-specific expression in mouse ES cells

We chose the ES cell serum/LIF system and analyzed 14 independent cell lines to robustly detect sex-specific differences. Because the ES cells in serum/LIF represent a less “naïve” state, with higher methylation levels, we reasoned that the transcriptomes from male and female cells would better reflect epigenetic differences dependent on the sex chromosome complement. In fact, we found many differentially expressed genes, including TFs belonging to the pluripotency network.

Together with results from previous reports, our data support the hypothesis that the sex chromosomes affect autosomal gene expression and epigenetic features, which can determine the sex-specific transcriptome later in development. In ES cells, the sex chromosome complement can influence gene expression before and after differentiation due to (1) gene activity from the Y chromosome in males and dosage differences in X-linked genes before X inactivation in females; (2) imprinted X-linked genes, active from the paternal, but not maternal, X chromosomes; (3) genes that escape XCI and are not dosage compensated when ES cells are differentiated; and (4) the process of XCI itself, which can substantially alter the availability and composition of epigenetic complexes in female relative to male cells. In addition, there is evidence that XCI per se delays the differentiation of female ES cells [27]. We suggest that this delay opens a window of opportunity for establishing epigenetic modifications unique to the female epigenome.

### Dosage of transcription factor *Prdm14* correlates with enhancer activity

In the light of the hypothesis, we focused on differentially expressed TFs and ERFs because they have the potential to imprint the epigenetic landscape and affect subsequent transcriptional activity. Because TFs often act synergistically or in multimers, variations in their dosage can quantitatively fine-tune the response of their target genes and can even expand the range of their targets. In addition, many epigenetic modifications are established by protein complexes and dosage differences in individual components can alter their stoichiometry or composition [92, 93]. In fact, our results indicate that the dosage of Prdm14 partially determines the activity of a developmentally regulated enhancer. This sequence, responsive to Oct4, Nanog, and Prdm14, induces higher transcriptional activity in the cells that have higher *Prdm14* levels, i.e., the female cells, even though *Oct4* and *Nanog* are not differentially expressed. As biological validation of regulatory elements progresses, it will be interesting to test the response of other sequences to the TF sex-specific biases.

Prdm14 can both activate and repress gene expression depending, at least partially, on its partner proteins [66]. Some targets of Prdm14 have been identified by siRNA knockdown [67] and gene knockout [71]. Higher levels of *Prdm14* in XX ES cells correlates with repression of *Dnmt3b, Cdkn1c*, *FoxA2,* and *Gata4* and activation of *Dazl*, *Mitf*, and *Zeb1*, indicating that dosage is a factor in regulation of some of its targets*.* Indeed, our luciferase assays identified a motif responsive to *Prdm14* dosage.

Not all genes exhibited the differences in response to Prdm14 predicted by the knockdown studies. Clearly, the relationship between levels of a TF and transcriptional outcome in vivo is not linear, and other factors, such as accessibility, motif numbers or combinations, are involved. Variations in the actual sequence of the motif might also contribute to the response to Prdm14.

### X-linked genes can be divided into four categories according to their expression levels

The X chromosome is the major contributor to female-enriched gene expression in the ES cells. Of the X-linked RNAs enriched in XX ES cells, there are two categories: one includes those expressed at a 2-fold level compared to the male cells, correlating with the dosage of the X chromosome. The other category encompasses X-linked genes that are upregulated *more* than 2-fold in the female cells. This indicates that these genes are influenced by an additional layer of regulation. For example, they could be responding to transcription factors that are more active in the female cells. Another possibility is that these genes are expressed more highly from one of the parental X chromosomes because it inherited a more open chromatin at its corresponding regulatory element. Yet a further explanation is that one of the two alleles of these genes is more highly expressed because of regulatory polymorphisms. Further studies to delineate the strain-specific transcriptional differences will help to answer this question.

The third, and most striking category, consists of X-linked genes that are expressed *equally* between male and female ES cells. Fifty percent of all expressed X-linked genes are in this group. This is not unique to our cells but is also seen in recent scRNA-seq data [75]. Considering that these genes are already dosage compensated, it will be interesting to determine the consequences of XCI on their relative levels.

A very limited number of X-linked genes constitute a fourth category. These are genes more highly expressed in male than in female ES cells, despite the presence of only one X chromosome. These may be responding directly or indirectly to genes expressed from the Y chromosome. Further studies will shed light on the mechanisms involved in this expression pattern.

### Differentiation markers are sex-specific in ES cells cultured in serum/LIF

A previous study comparing the transcriptomes of ES cells cultured in serum/LIF versus 2i found that whereas the pluripotency genes were expressed at similar levels in both conditions, cells in serum/LIF exhibited higher expression of differentiation markers [94]. This is because these cells are in a metastable condition, balanced between pluripotency and lineage determination [30]. Interestingly, our data show that ES cells express certain differentiation markers, but they also highlight that male and female cell lines exhibit distinct profiles. It will be exciting to elucidate how the sex-specific regulatory networks connect to the dimorphisms in lineage determining molecules.

### Comparison with single-cell RNA-seq and human data

Our data correlated well with a comparable dataset generated from single-cell RNA-seq [75]. Many differences were observed, however, especially for genes expressed at very low levels. Because transcription occurs in bursts, single-cell expression analysis more closely models the dynamics of RNA pol II activity in each individual cell [95–99]. Master transcriptional factors, however, are generally expressed at low levels. Thus, to determine the presence or absence of a TF, and especially to compare between different cells or tissues, we suggest that bulk RNA extraction and sequencing gives a more robust picture of the expression profile.

Sex biases in gene expression have been reported in human ES cells with microarrays [29]. However, undifferentiated human female ES cells have already undergone XCI and show little overlap with our datasets. Whether this is because of the X chromosome status or species-specific developmental differences (or both) is not clear, but it emphasizes that comparisons between ES cells of divergent species will be necessary.

### Levels of some imprinted genes are sex-specific

We revealed a layer of regulation of imprinted genes beyond the differential DNA methylation that maintains monoallelic expression. Even though only the maternal alleles of *Cdkn1c*, *Phlda2*, and *Slc22a18* were active, they expressed higher RNA levels in the XY cells than in the XX cells, an observation that will be followed up to determine the additional factors regulating these genes.

### Sex chromosome-dependent transcriptional regulation

A previous report comparing global expression analysis in four somatic tissues of adult 40,XX and 39,X by microarray had identified genes overexpressed in XX females and revealed transcriptional changes in autosomal genes in response to X chromosome dosage [100]. Comparisons between XX, XY, and XO ES cells allowed us to stratify the transcripts with expression dependent on the presence or absence of the X and Y chromosomes in early development. Genes with sex biases in somatic tissues do not overlap with our data, likely due to the difference in transcriptomes between embryonic and differentiated cells. The XO ES cells are much more similar to XY cells, supporting the previously posited hypothesis that the presence of two X chromosomes delays differentiation due to the need for XCI [27]. Intriguingly, the XO ES cells also exhibited down-regulation of two imprinted genes, the maternally expressed *Grb10* and *Rian* genes*.*

We also found evidence that the absence of two X chromosomes leads to overexpression of genes associated with Turner syndrome, including *Fgfr3* and *Wnt4* [88]. However, there are differences between 39,X mice and Turner syndrome patients, with mice presenting a milder phenotype [101]. A recent comparative study of DNA methylation and gene expression differences between 46,XX and 45,X females performed in leukocytes showed no overlap with the differentially expressed genes in our dataset [102], likely because of species differences and because none of the reported genes are expressed during embryogenesis.

Many of the regulatory elements that direct tissue-specific expression have a “poised” chromatin status in early development, i.e., they exhibit both activating and repressing chromatin marks that are “resolved” upon differentiation in one way or another [103]. Thus, epigenetic marks can be latent, with the potential to result in transcriptional outcomes only later in the life of the organism. The same scenario holds for TFs that work in concert to activate gene expression, where one serves as a placeholder until a partner or co-factor appears later on [104]. Evidence is accumulating that disruptions in embryonic stages can have consequences that only become apparent in adults [105, 106]. Integration of our results with previously reported datasets provides insights into how sex-specific differences appear and are translated into later developmental events [107, 108]. In addition, our data will contribute to understanding how male and female embryos differ in their response to the environment, with implications for the impact of maternal behaviors on fetal development.

## Conclusions

Sex biases in gene expression lead to critical, health-related sexual dimorphisms that are widely appreciated but still understudied. We show here that differential expression patterns are established in early embryogenesis, before hormonal influence is unleashed. At the level of pathway analysis, these expression differences integrate distinct networks and are dependent directly or indirectly on the sex chromosome complement. Thus, substantial contributions to sex-related differences occur prior to and possibly upstream of gonadal sex determination [109].

Our datasets from XO ES cell lines are an important platform for understanding the impact of sex chromosome aneuploidies on pre-implantation embryogenesis and lineage determination. They will also contribute to refining the direct and indirect mechanisms by which the sex chromosomes interact with the autosomal component of the genome.

## Additional files

Additional file 1: Figure S1. Derivation of mouse ES cell lines. F1 hybrid blastocysts were obtained at embryonic day 3.5 from reciprocal crosses of mouse substrains C57BL/6 and CAST/EIJ (designated as B and C, respectively). Blastocysts were cultured individually and used to establish independent cell lines.

Additional file 2: Table S1. Real-time PCR primers.

Additional file 3: Table S2. Transcription factor motifs enriched in promoters of differentially expressed genes in female and male ES cells.

Additional file 4: Table S3. Catalog of transcription factors (TFs) and epigenetic and remodeling factors (ERFs) expressed differentially in female and male ES cells.

Additional file 5: Figure S2. Identification of candidate regulatory elements. (A) Top, graphic display of the conservation profiles for regions upstream of *Zfx* from Dcode.org [64, 110]. The base genome is mouse. Evolutionarily conserved regions (ECRs) of a minimum of 100 bp conserved above 70% sequence identity are displayed as red (intergenic) peaks, with the x-axis representing positions in the base genome and the y-axis representing percentage identity between the base and the aligned genomes. Predicted transcription factor motifs are depicted as colored bars. Arrowhead points to predicted motif of TF expressed more highly in female ES cells. Bottom, UCSC genome browser view of the same regions including histone modifications from ENCODE data in mouse ES cells (http://genome.ucsc.edu, NCBI37/mm9). (B) Conservation analysis, TF motif prediction and UCSC browser view as in (A) for the *Tcf3* gene. Arrowhead points to predicted motif of TF expressed more highly in male ES cells. (C) Conservation analysis, TF motif prediction and UCSC browser view as in (A) for the *Apln* gene, with arrowheads indicating motifs predicted to bind TFs more highly expressed in male ES cells.

Additional file 6: Table S4. Expression in undifferentiated murine embryonic stem (ES) cells of genes that escape X chromosome inactivation (XCI) after differentiation (BC cell lines).

Additional file 7: Table S5. Examples of genes expressed in undifferentiated ES cells of genes that do not escape XCI (BC cell lines).

Additional file 8: Figure S3. Allele-specific expression analysis for imprinted gene *Cdkn1c*. RT-PCR was followed by allele-specific restriction digest of the *Cdkn1c* coding sequence and polyacrylamide gel analysis. A single nucleotide polymorphism in the *M. castaneus* allele generates a restriction site for *aTaq*I. Two different F1 hybrid ES cell lines each derived from reciprocal crosses of C57BL/6 (B) and CAST/EIJ (C) mice exhibited monoallelic (BxC) and biased expression (CxB) from the maternal allele. The first lane is the marker, the last two lanes are digested controls from PCR products from B and C genomic DNA.

Additional file 9: Figure S4. Comparison of expression differences for 14 selected genes from our dataset with a single-cell (sc) RNA-seq study [75] shows a high degree of correlation. Individual plots of expression from the scRNA-seq analysis shows the variability within the assay and the abundance of zero readouts for some genes. To avoid this confounder, a sign test was performed using a binomial exact test which affirmed the sex-specific biases seen within our dataset.

Additional file 10: Table S6. Genes with a sex-specific bias that overlap between ES cells and 5.5 dpc embryos.

Additional file 11: Figure S5. Chromosomal distribution of genes enriched in BCO when compared to BCF ES cells. BCO ES cell lines had enrichment of 423 genes, 49% of which overlapped with genes enriched in BCM relative to BCF (FDR < 0.01). The genes common to both BCO and BCM had statistically higher enrichment relative to BCF, averaging 2.55- versus 2.23-fold (students t-test, two tailed *p* < 0.001). Error bars denote standard deviation.

## Abbreviations

BCM, BCF: F1 hybrid male and female ES cell lines resulting from a C57BL/6 female × CAST/EiJ male cross
BCO: F1 hybrid XO ES cell line from the same cross
CBM, CBF: F1 hybrid male and female ES cell lines resulting from a CAST/EiJ female × C57BL/6 male cross
CI: Confidence interval
ERFs: Epigenetic and remodeling factors
ES: Embryonic stem
FDR: False discovery rate
LIF: Leukemia inhibitory factor
lncRNAs: Long non-coding RNAs
MEFs: Mouse embryonic fibroblasts
PAGE: Polyacrylamide gel electrophoresis
TFs: Transcription factors
XCI: X chromosome inactivation
