# Supplementary figures and images for: Sex chromosomes drive gene expression and regulatory dimorphisms in mouse embryonic stem cells
Source: Biol Sex Differ. 2017 Aug 17;8:28. doi: 10.1186/s13293-017-0150-x (PMC5561606; doi:10.1186/s13293-017-0150-x)

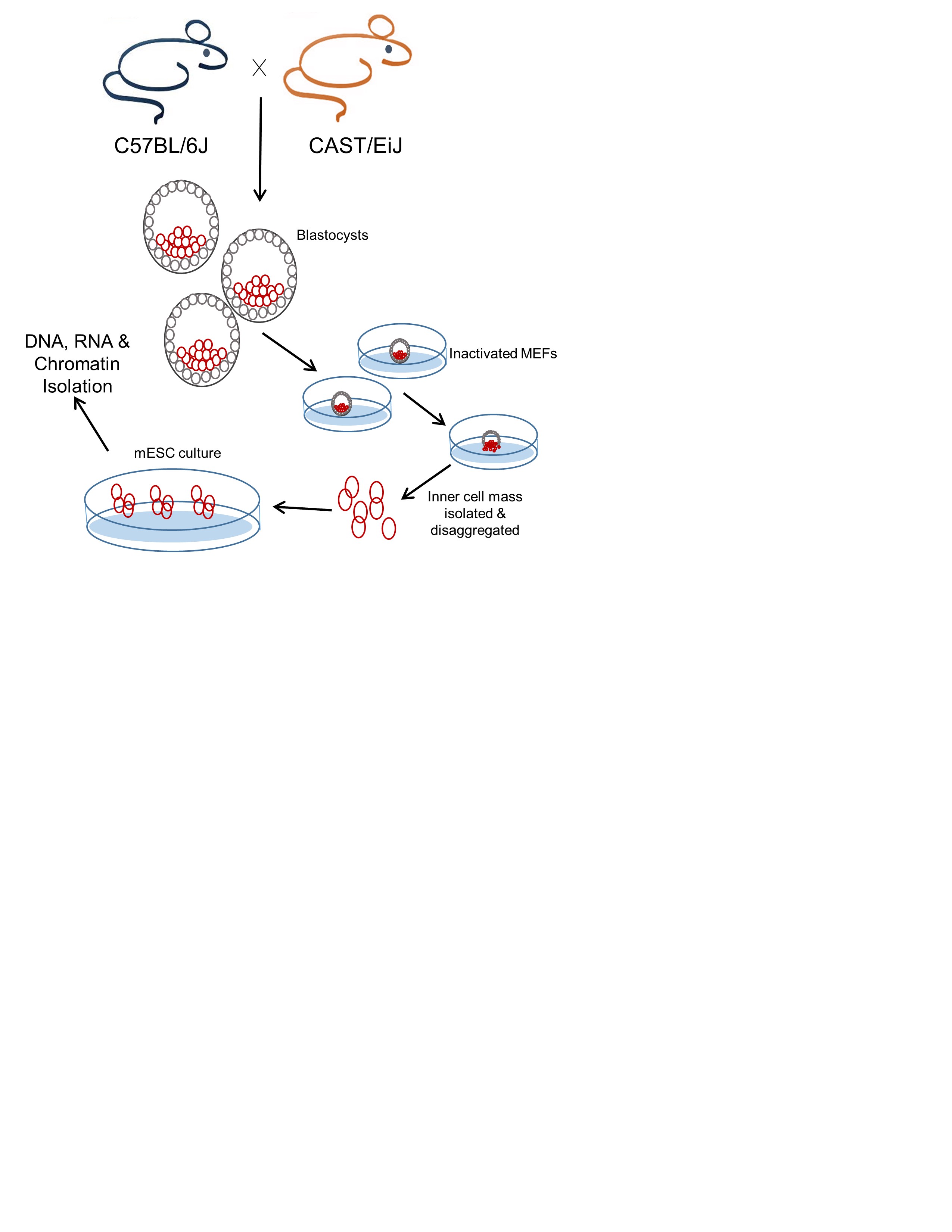

Supplement: Supplementary file 1 — Derivation of mouse ES cell lines. F1 hybrid blastocysts were obtained at embryonic day 3.5 from reciprocal crosses of mouse substrains C57BL/6 and CAST/EIJ (designated as B and C, respectively). Blastocysts were cultured individually and used to establish independent cell lines. [file 13293_2017_150_MOESM1_ESM.jpg]

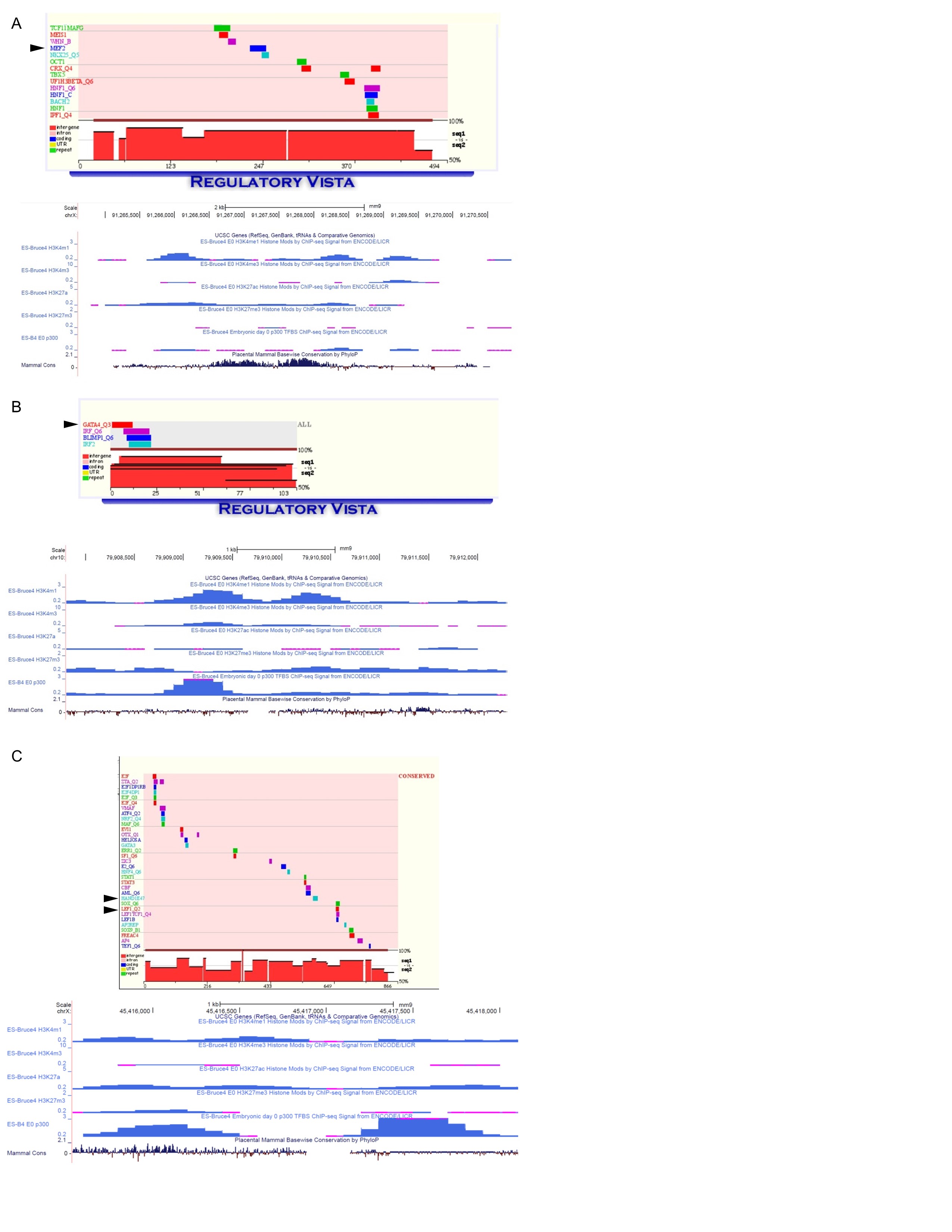

Supplement: Supplementary file 5 — Identification of candidate regulatory elements. (A) Top, graphic display of the conservation profiles for regions upstream of Zfx from Dcode.org [64, 110]. The base genome is mouse. Evolutionarily conserved regions (ECRs) of a minimum of 100 bp conserved above 70% sequence identity are displayed as red (intergenic) peaks, with the x-axis representing positions in the base genome and the y-axis representing percentage identity between the base and the aligned genomes. Predicted transcription factor motifs are depicted as colored bars. Arrowhead points to predicted motif of TF expressed more highly in female ES cells. Bottom, UCSC genome browser view of the same regions including histone modifications from ENCODE data in mouse ES cells (http://genome.ucsc.edu, NCBI37/mm9). (B) Conservation analysis, TF motif prediction and UCSC browser view as in (A) for the Tcf3 gene. Arrowhead points to predicted motif of TF expressed more highly in male ES cells. (C) Conservation analysis, TF motif prediction and UCSC browser view as in (A) for the Apln gene, with arrowheads indicating motifs predicted to bind TFs more highly expressed in male ES cells. [file 13293_2017_150_MOESM5_ESM.jpg]

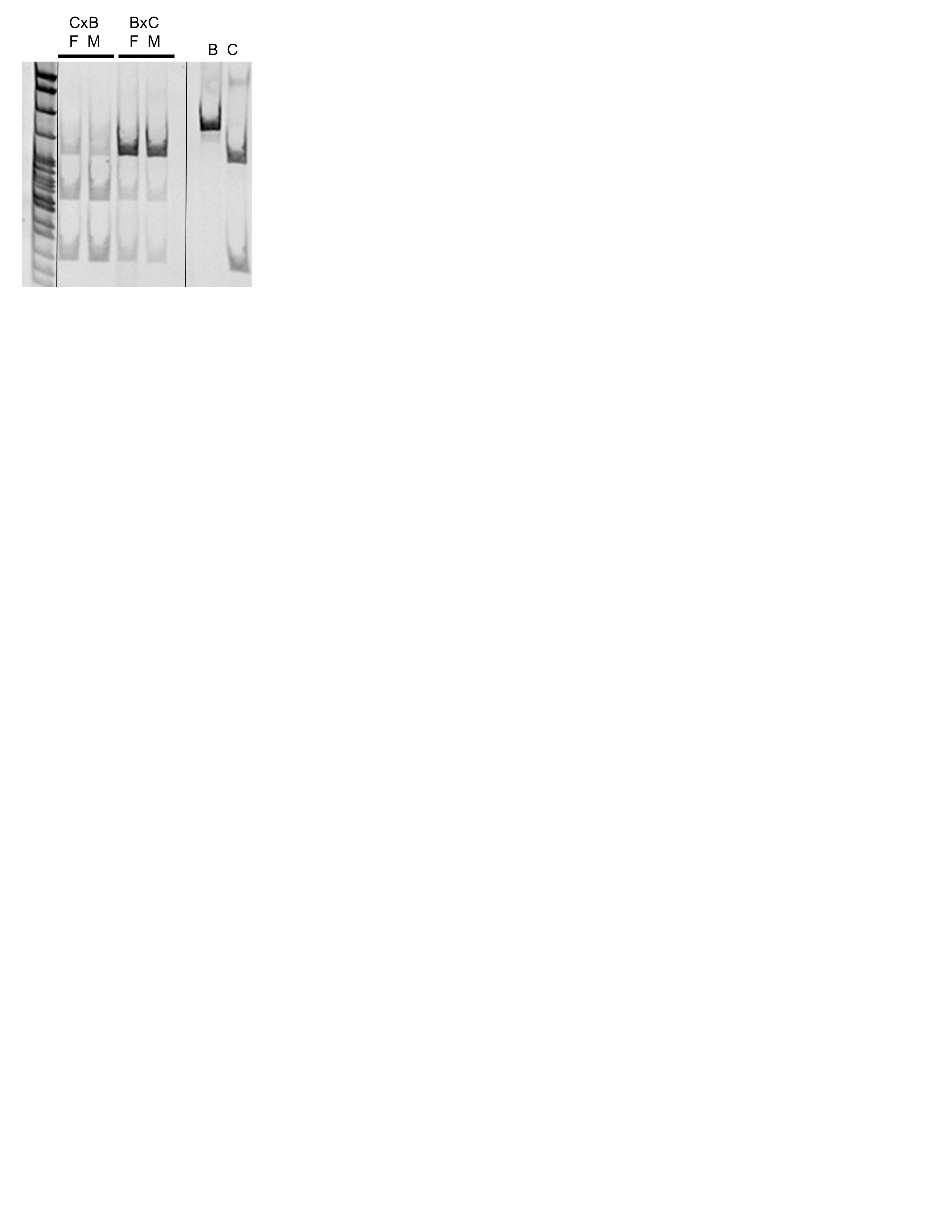

Supplement: Supplementary file 8 — Allele-specific expression analysis for imprinted gene Cdkn1c. RT-PCR was followed by allele-specific restriction digest of the Cdkn1c coding sequence and polyacrylamide gel analysis. A single nucleotide polymorphism in the M. castaneus allele generates a restriction site for aTaqI. Two different F1 hybrid ES cell lines each derived from reciprocal crosses of C57BL/6 (B) and CAST/EIJ (C) mice exhibited monoallelic (BxC) and biased expression (CxB) from the maternal allele. The first lane is the marker, the last two lanes are digested controls from PCR products from B and C genomic DNA. [file 13293_2017_150_MOESM8_ESM.jpg]

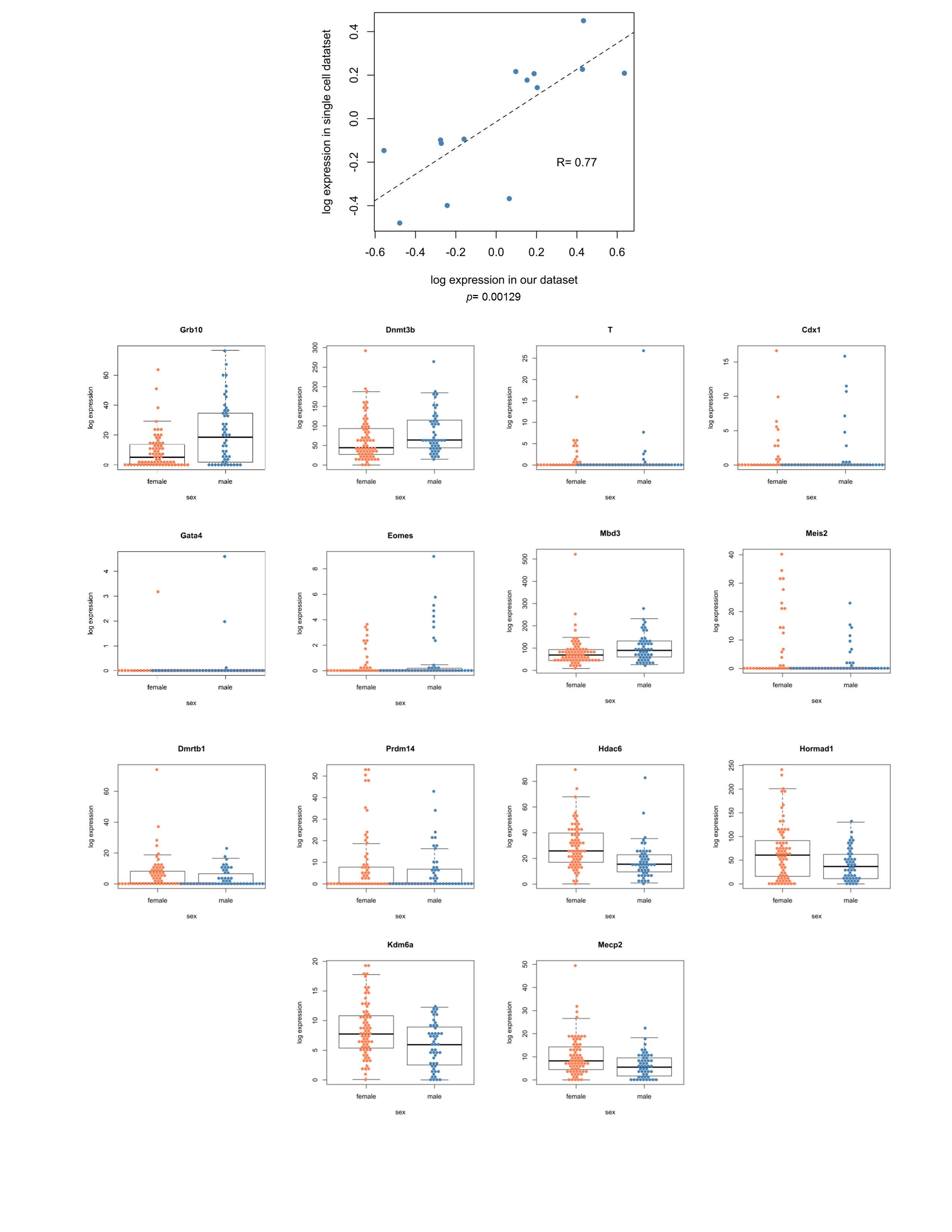

Supplement: Supplementary file 9 — Comparison of expression differences for 14 selected genes from our dataset with a single-cell (sc) RNA-seq study [75] shows a high degree of correlation. Individual plots of expression from the scRNA-seq analysis shows the variability within the assay and the abundance of zero readouts for some genes. To avoid this confounder, a sign test was performed using a binomial exact test which affirmed the sex-specific biases seen within our dataset. [file 13293_2017_150_MOESM9_ESM.jpg]

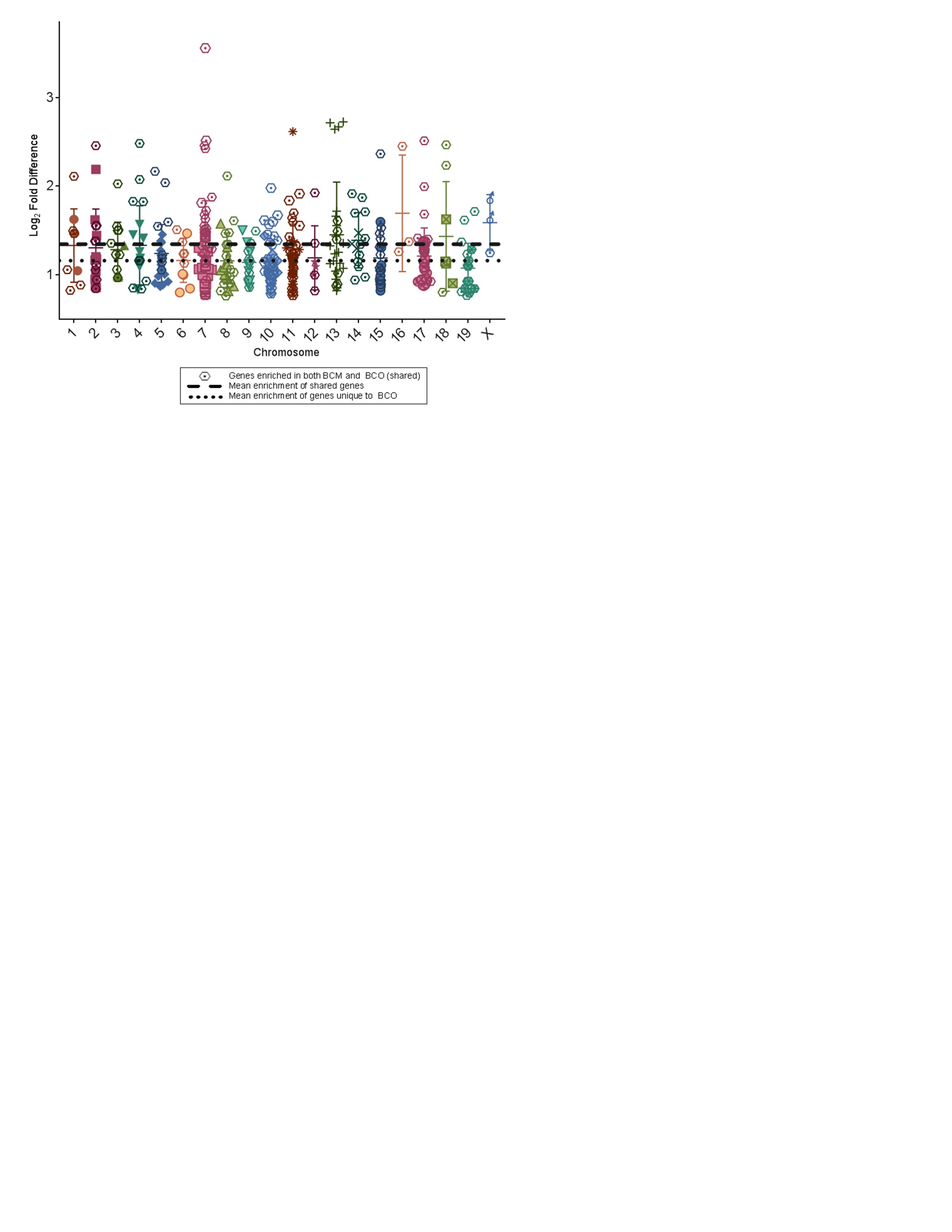

Supplement: Supplementary file 11 — Chromosomal distribution of genes enriched in BCO when compared to BCF ES cells. BCO ES cell lines had enrichment of 423 genes, 49% of which overlapped with genes enriched in BCM relative to BCF (FDR < 0.01). The genes common to both BCO and BCM had statistically higher enrichment relative to BCF, averaging 2.55- versus 2.23-fold (students t-test, two tailed p < 0.001). Error bars denote standard deviation. [file 13293_2017_150_MOESM11_ESM.jpg]
