# Supplementary material for: Sex chromosomes drive gene expression and regulatory dimorphisms in mouse embryonic stem cells
Source: Biol Sex Differ. 2017 Aug 17;8:28. doi: 10.1186/s13293-017-0150-x (PMC5561606; doi:10.1186/s13293-017-0150-x)
Supplement: Supplementary file 2 — Real-time PCR primers. [file 13293_2017_150_MOESM2_ESM.docx]

Additional Table 1. Real-Time PCR Primers

| **Gene** | **Forward** | **Reverse** |
| --- | --- | --- |
| *Grb10* | TGACAACAGCTGGACTCTGG | TCTTCCAAGACTTGCGTCCT |
| *Dnmt3b* | ACGCAGGACATGACAGGAGAT | AGAATATCAGAGCCATTCCCATCA |
| *T* | GTCAAACTCACCAACAAGCTCAA | GAATCCGAGGTTCATACTTATGCA |
| *Eomes* | CGCCCACTACAATGTTTTCGT | CTGGGTGAACGTACATCTTATTGC |
| *Cdx1* | GCGGTGGCAGCGGTAAG | GGCGTTGGTGGTCTGTGTAGA |
| *Gata4* | CCTGTGCCAACTGCCAGACT | CTCCTTCCGCATTGCAAGAG |
| *Mbd3* | AAGCAACCGGTGACCAAGAT | CAATGTCAAAGGCACTCAATCC |
| *Meis2* | CCTCAAACCCAGAGCTGGATAA | AGCTAATGTACCGGTGGCAGAA |
| *Dmrtb1* | CCCTACCAGTCCTTTCCACTTTC | AGGTCTCTGGCTGGCTCTGA |
| *Prdm14* | CGGCCCTACCTGTGTTCAA | ATGTGCTTGTTCAGGCTGGAA |
| *Hdac6* | CCTGGACTCTGAGCTCCTTACC | TCTGTGGTGCGGAGATGCT |
| *Hormad1* | CAGGCGGTGCGTAAAAAAA | CCAATGCACTCAGGGAAGCT |
| *Kdm6a* | GGCACGGGCGGACAA | AGATCAAAAACCTCCACCTCACA |
| *Mecp2* | AAAGGAAGTCTGGCCGATCTG | CATTAGGGTCCAAGGAGGTGTCT |
