# Supplementary material for: Sex chromosomes drive gene expression and regulatory dimorphisms in mouse embryonic stem cells
Source: Biol Sex Differ. 2017 Aug 17;8:28. doi: 10.1186/s13293-017-0150-x (PMC5561606; doi:10.1186/s13293-017-0150-x)
Supplement: Supplementary file 3 — Transcription factor motifs enriched in promoters of differentially expressed genes in female and male ES cells. [file 13293_2017_150_MOESM3_ESM.docx]

Additional Table 2. Transcription Factor Motifs Enriched in Promoters of Differentially Expressed Genes in Female and Male ES cells.

| **XX enriched** |  |  |  |  | **XY enriched** |  |  |  |
| --- | --- | --- | --- | --- | --- | --- | --- | --- |
| -1500 to +500 |  | -5000 to +500 |  |  | -1500 to +500 |  | -5000 to +500 |  |
| homer | p-value | homer | p-value |  | homer | p-value | homer | p-value |
| Arid5a | 1xe-14 | Irf4 | 1xe-11 |  | Elk4 | 1xe-12 | Pitx1 | 1xe-14 |
| E2f2 | 1xe-13 | Mef2c | 1xe-10 |  | Runx1 | 1xe-12 | Nr5a2 | 1xe-12 |
| Myc | 1xe-12 | Zscan4 | 1xe-8 |  | Foxq1 | 1xe-11 | Nkx3-1 | 1xe-9 |
| Mafb | 1xe-12 | Gfi1 | 1xe-8 |  | Olig2 | 1xe-10 | Brca1 | 1xe-9 |
| Nkx2-5 | 1xe-12 | Mecom | 1xe-7 |  | Hic1 | 1xe-9 | Prrx2 | 1xe-9 |
| HoxA2 | 1xe-10 | Zbtb7b | 1xe-6 |  | Irf5 | 1xe-9 | Tcf3 | 1xe-8 |
| Tead2 | 1xe-9 | Zfx | 1xe-5 |  |  |  | Bcl6 | 1xe-8 |
| Smad3 | 1xe-8 |  |  |  |  |  |  |  |
| HoxA5 | 1xe-8 |  |  |  |  |  |  |  |
