# Supplementary material for: Sex chromosomes drive gene expression and regulatory dimorphisms in mouse embryonic stem cells
Source: Biol Sex Differ. 2017 Aug 17;8:28. doi: 10.1186/s13293-017-0150-x (PMC5561606; doi:10.1186/s13293-017-0150-x)
Supplement: Supplementary file 4 — Catalog of transcription factors (TFs) and epigenetic and remodeling factors (ERFs) expressed differentially in female and male ES cells. [file 13293_2017_150_MOESM4_ESM.docx]

Additional Table 3. Catalog of Transcription Factors (TFs) and Epigenetic and Remodelling Factors (ERFs) expressed differentially in Female and Male ES cells.

| **XX-enriched TFs** | **XX-enriched ERFs** |  | **XY-enriched TFs** | **XY-enriched ERFs** |
| --- | --- | --- | --- | --- |
| Aff2 | Apobec2 |  | Arid1a | 0610010K14Rik |
| Dmrtb1 | Atrx |  | Arid3a | Anp32a |
| Elf4 | Aurkc |  | Bcl6b | Arrb1 |
| Elk3 | Baz2b |  | Cdx1 | Cdk2 |
| Foxc2 | Bmi1 |  | Cdx2 | Cxxc1 |
| Hmga2 | Brcc3 |  | Creb3l3 | Dapk3 |
| Hmgb3 | Dzip3 |  | E2f7 | Dnmt3a |
| Hoxa10 | Eid1 |  | Egr4 | Dnmt3b |
| Hoxa2 | Hmgn3 |  | Eomes | Dnmt3l |
| Hoxa5 | Huwe1 |  | Evx1 | Dot1l |
| Hoxb2 | Kdm6a |  | Fos | Ehmt2 |
| Hoxb6 | Mecp2 |  | Foxh1 | Hdac5 |
| Hoxb7 | Nap1l2 |  | Foxi3 | Hlcs |
| Hoxb9 | Ogt |  | Foxp4 | Mbd3 |
| Hoxc6 | Parp3 |  | Gata4 | Mdc1 |
| Hoxc8 | Prkca |  | Hand1 | Phc1 |
| Hoxd10 | Rps6ka3 |  | Hlf | Smarcd1 |
| Hoxd13 | Taf1 |  | Hmga1-rs1 | Tada3 |
| Klf8 | Tdrd7 |  | Hmgxb4 | Ubn1 |
| Mbtps2 | Top2b |  | Insm1 | Uty |
| Mecom | Trim16 |  | Irf8 |  |
| Meis2 | Zmym3 |  | Junb |  |
| Mitf |  |  | Lef1 |  |
| Nfia |  |  | Lin28a |  |
| Obox6 |  |  | Mef2b |  |
| Prdm14 |  |  | Mesp1 |  |
| Prrx1 |  |  | Mixl1 |  |
| Rhox1 |  |  | Mycn |  |
| Rhox6 |  |  | Ncor2 |  |
| Rhox9 |  |  | Nfkb2 |  |
| Runx1 |  |  | Nfxl1 |  |
| Sohlh2 |  |  | Nkx1-2 |  |
| Spic |  |  | Nr6a1 |  |
| Tbx15 |  |  | Olig1 |  |
| Tulp4 |  |  | Pbx2 |  |
| Zbtb4 |  |  | Pou2f3 |  |
| Zbtb7c |  |  | Pou3f1 |  |
| Zeb1 |  |  | Prdm6 |  |
| Zeb2 |  |  | Rara |  |
| Zfhx4 |  |  | Relb |  |
| Zfp112 |  |  | Sfpi1 |  |
| Zfp182 |  |  | Six2 |  |
| Zfp275 |  |  | Sox11 |  |
| Zfp3 |  |  | Sp5 |  |
| Zfp449 |  |  | Sp8 |  |
| Zfp59 |  |  | T |  |
| Zfp9 |  |  | Tcf3 |  |
| Zfx |  |  | Tcf7 |  |
| Zxdb |  |  | Thap3 |  |
|  |  |  | Tulp3 |  |
|  |  |  | Wiz |  |
|  |  |  | Ybx2 |  |
|  |  |  | Zbtb12 |  |
|  |  |  | Zbtb7a |  |
|  |  |  | Zfp296 |  |
|  |  |  | Zfp523 |  |
|  |  |  | Zfp647 |  |
|  |  |  | Zglp1 |  |
|  |  |  | Zmiz2 |  |
