# Supplementary material for: Sex chromosomes drive gene expression and regulatory dimorphisms in mouse embryonic stem cells
Source: Biol Sex Differ. 2017 Aug 17;8:28. doi: 10.1186/s13293-017-0150-x (PMC5561606; doi:10.1186/s13293-017-0150-x)
Supplement: Supplementary file 6 — Expression in undifferentiated murine embryonic stem (ES) cells of genes that escape X chromosome inactivation (XCI) after differentiation (BC cell lines). [file 13293_2017_150_MOESM6_ESM.docx]

Additional Table 4. Examples of genes expressed in undifferentiated ES cells of genes and that do not escape XCI (BC cell lines). Several X-linked genes exhibited increased expression among the XX lines that was well above the levels detected in either XY or XO. Among the genes showing the highest degree of difference between XX and either XY or XO were a number of genes within the X-linked lymphocyte regulated (*Xlr)* gene family. The difference in expression based on sex chromosome composition and the associated false discovery rate is shown above along with chromosomal location.

| **Mouse Gene Symbol** | **XX vs. XY** | | **XX vs. XO** | | **Position (mm10)** |
| --- | --- | --- | --- | --- | --- |
|  | **Fold change** | **FDR** | **Fold change** | **FDR** |  |
|  |  |  |  |  |  |
| *Rhox1* | 5.5 | 0.0014 | 13.5 | 0.0026 | chrX:37213805-37222258 |
| *Gm9* | 2.7 | 0.0176 | 3.8 | 0.0182 | chrX:37208502-37211041 |
| *Xlr3a* | 87.5 | 0.0014 | 241.8 | 0.0316 | chrX:73086293-73097095 |
| *Xlr* | 5.9 | 0.0125 | 4.4 | 0.0321 | chrX:73148841-73158399 |
| *Xlr3c* | 32.4 | 0.0014 | 105.5 | 0.0275 | chrX:73254540-73265390 |
| *Nxf3* | 12.6 | 0.0014 | 19.2 | 0.0036 | chrX:136072099-136085255 |
