# Supplementary material for: Sex chromosomes drive gene expression and regulatory dimorphisms in mouse embryonic stem cells
Source: Biol Sex Differ. 2017 Aug 17;8:28. doi: 10.1186/s13293-017-0150-x (PMC5561606; doi:10.1186/s13293-017-0150-x)
Supplement: Supplementary file 7 — Examples of genes expressed in undifferentiated ES cells of genes that do not escape XCI (BC cell lines). [file 13293_2017_150_MOESM7_ESM.docx]

Additional Table 5. Expression in undifferentiated murine embryonic stem (ES) cells of genes that escape X chromosome inactivation (XCI) after differentiation (BC cell lines). Genes previously identified to escape X-inactivation [26] were isolated from the expression data generated by RNA-sequencing. The difference in expression based on sex-chromosome composition and the associated false discovery rate is shown above along with chromosomal location.

| **Mouse Gene Symbol** | **XX vs. XY** | | **XX vs. XO** | | **Position (mm10)** |
| --- | --- | --- | --- | --- | --- |
|  | **Fold change** | **FDR** | **Fold change** | **FDR** |  |
|  |  |  |  |  |  |
| *Clcn5* | 2.0 | 0.0014 | 1.7 | 0.0212 | chrX:7158412-7319358 |
| *Bgn* | 5.7 | 0.0014 | 3.8 | 0.0014 | chrX:73483601-73495936 |
| *Taf1* | 2.0 | 0.0014 | 1.7 | 0.0063 | chrX:101532735-101601789 |
| *Chic1* | 2.0 | 0.0014 | 2.0 | 0.0014 | chrX:103356476-103396118 |
| *Rnf128* | 1.6 | 0.0386 | 2.5 | 0.0014 | chrX:139610620-139673145 |
| *Ftx* | 1.8 | 0.0425 | 2.0 | 0.0229 | chrX:103560910-103616954 |
| *Sh3bgrl* | 3.6 | 0.0014 | 4.0 | 0.0014 | chrX:109095407-109162467 |
| *Gprasp1* | 1.6 | 0.0218 | 2.0 | 0.0014 | chrX:135742692-135803468 |
| *Trappc2* | 1.9 | 0.0111 | 2.4 | 0.0014 | chrX:166440755-166453140 |
