# Supplementary material for: Sex chromosomes drive gene expression and regulatory dimorphisms in mouse embryonic stem cells
Source: Biol Sex Differ. 2017 Aug 17;8:28. doi: 10.1186/s13293-017-0150-x (PMC5561606; doi:10.1186/s13293-017-0150-x)
Supplement: Supplementary file 10 — Genes with a sex-specific bias that overlap between ES cells and 5.5 dpc embryos. [file 13293_2017_150_MOESM10_ESM.docx]

Additional Table X. Genes with a sex-specific bias that overlap between ES cells

and 5.5 dpc embryos.

| **XX Enriched** | **XY Enriched** |
| --- | --- |
| Capn2 | Anp32a |
| Cd63 | Fkbp4 |
| Itga7 | Ppp1r10 |
| Xbp1 | Thop1 |
| Sparc | Anp32b |
| AI662270 | Dnmt3b |
| Hist1h2bc | Crmp1 |
| Ctsl | Ddx3y |
| Dab2 | Uty |
| Lgals1 | Ncan |
| Apom | Anp32a |
| Mcfd2 |  |
| Cd59a |  |
| B2m |  |
| Commd3 |  |
| Gsn |  |
| Anxa5 |  |
| S100a4 |  |
| S100a6 |  |
| Mtus2 |  |
| Gng12 |  |
| Aqp8 |  |
| Htra1 |  |
| Igf2 |  |
| Serpinh1 |  |
| Col4a1 |  |
| Itgb3 |  |
| Sphk1 |  |
| Fam161a |  |
| Havcr2 |  |
| Fbxw10 |  |
| Col1a1 |  |
| Fam110c |  |
| Nid1 |  |
| Pxdc1 |  |
| Cxcl14 |  |
| Gjb2 |  |
| Lynx1 |  |
| Sytl3 |  |
| Chsy3 |  |
| Anxa1 |  |
| Mlana |  |
| Sh3pxd2a |  |
| Gpat2 |  |
| Atrn |  |
| Angpt4 |  |
| Creb3l1 |  |
| Tm4sf1 |  |
| Cdkn2b |  |
| Klb |  |
| Emp1 |  |
| Galnt18 |  |
| Mical2 |  |
| Emp3 |  |
| Anxa2 |  |
| Adam10 |  |
| Ctsh |  |
| Xist |  |
| Wbp5 |  |
| Trap1a |  |
| Nup62cl |  |
| Trappc2 |  |
| Efhc2 |  |
| Rhox6 |  |
| Fmr1nb |  |
| Pnma5 |  |
| Xlr3a |  |
| Xlr3b |  |
| Xlr3c |  |
| Bgn |  |
| Dusp9 |  |
| Slc6a8 |  |
| Efnb1 |  |
